# The Anticonvulsant Effect of Long-Term Valproate Might Be Attributable to Altered Expression of Selected Genes in Mice

**DOI:** 10.3390/ijms27125281

**Published:** 2026-06-10

**Authors:** Monika Banach, Przemysław Kołodziej, Jacek Bogucki, Kinga Borowicz, Anna Bogucka-Kocka

**Affiliations:** 1 Independent Experimental Neuropathophysiology Unit, Department of Toxicology, Faculty of Pharmacy, Medical University of Lublin, Jaczewskiego 8b Street, 20-090 Lublin, Poland; monika.banach@umlub.edu.pl; 2 Department of Biology and Genetics, Faculty of Pharmacy, Medical University of Lublin, 4A Chodzki Street, 20-093 Lublin, Poland; anna.bogucka-kocka@umlub.edu.pl; 3 Department of Research Methodology in Medicine, Institute of Medical Biology, Faculty of Medicine, The John Paul II Catholic University of Lublin, Konstantynów 1H St., 20-708 Lublin, Poland; jacek.bogucki@kul.pl

**Keywords:** valproate, antiepileptic drugs, chronic treatment, epilepsy, maximal electroshock-induced seizures, gene expression

## Abstract

In this study, we assessed the impact of repeated valproate administration on its anticonvulsant effects and side effects in mice. We measured the plasma and brain concentrations of valproate and examined changes in the expression of selected genes in the mouse hippocampus after both acute and chronic treatments. Electroconvulsions were induced using an alternating current (50 Hz, 25 mA, 0.2 s) through ear clip electrodes. Motor impairment and long-term memory deficits were assessed with the chimney test and passive avoidance task. Valproate concentrations in the brain and plasma were measured by a fluorescence polarization immunoassay. mRNA was isolated using a modified Chomczyński and Sacchi method, and RQ-PCR was performed with an Applied Biosystems 7900 using SDS and RQ Study software. The 50% effective dose (ED_50_) of valproate in the 14 × 2 protocol was significantly lower than the control. Despite no observed memory deficits in chronic protocols, the 50% toxic dose (TD_50_) for motor impairment was also significantly lower. Chronic valproate treatment did not alter the plasma and brain concentrations. However, the expression levels of three genes (CACNA1G, GAD1, SCN1A) were significantly higher in the chronic protocols with the higher dose of valproate compared to single protocols, suggesting a dose-dependent effect. The repeated administration of valproate resulted in both enhanced efficacy and increased toxicity in terms of motor impairment. The observed effect may be associated with transcriptional adaptations potentially mediated by epigenetic mechanisms rather than with pharmacokinetic events. To enhance the reliability of the results obtained in animal epilepsy models, antiepileptic drugs should be administered chronically.

## 1. Introduction

Pharmacotherapy is a standard method of the initial treatment of epilepsy. Prior to the implementation of bi- and polytherapy, it is recommended that single-drug treatment be attempted. Unfortunately, the first antiepileptic drug (AED) is effective in less than 50% of patients. The second one, with a different mechanism of action, frees another 13% of patients from seizures [1,2]. Polytherapy, in contrast to monotherapy, is often complicated by drug–drug interactions and toxicity without substantial improvement in outcome. The lower cost of therapy and the simplicity of dosage improve the compliance of monotherapy, which is fundamental to successful therapy.

Valproate (valproic acid, commonly used as its sodium salt, VPA), primarily used as a solvent, was serendipitously discovered as a potent anticonvulsant by Pierre Eymard in 1962 [3,4]. Nowadays, despite the availability of new generations of drugs, it is still prescribed for epileptic and non-epileptic conditions. VPA is indicated in different types of epilepsy, including generalized tonic–clonic seizures (as a first-line monotherapy or add-on treatment with the exception of women of childbearing age), tonic or atonic seizures, idiopathic generalized epilepsies, myoclonic seizures, Lennox–Gastaut syndrome, and Dravet syndrome (all six as a first-line treatment), partial seizures (second-line add-on treatment), absence seizures (second-line monotherapy or add-on treatment), and post-traumatic epilepsy. Parenteral preparations of VPA are also used as a second-line treatment for status epilepticus. Furthermore, this AED is employed in the treatment of certain psychiatric diseases, including schizophrenia and bipolar affective disorder [5,6,7,8].

This wide range of indications was extensively documented in preclinical studies. In experimental seizures, VPA displays anticonvulsant activity in the maximal electroshock test in mice (the most adequate model of tonic–clonic seizures), partial and secondarily generalized convulsions in amygdala-kindled rats, and pentylenetetrazole-induced clonic seizures in mice [9,10,11,12].

The multiplicity of mechanisms of action of VPA can justify its almost universal employment as an AED. The most important are: potentiation of GABA-ergic transmission, interaction with voltage-gated sodium channels, T-type calcium channels, modulation of NMDA receptor signaling, modulation of dopamine and serotonin transmission, and inhibition of histone deacetylase [3,10,13,14,15]. Furthermore, VPA is postulated to induce genomic alterations that could exert long-term effects on brain plasticity and anticonvulsant therapy [16].

The effectiveness of pharmacotherapy may alter during extensive exposure to a specific drug. Both the pharmacokinetic and pharmacodynamic nature of this phenomenon is possible. AEDs may induce or inhibit liver enzymes, which may lead to their autoinduction and/or affect the metabolism of other drugs. These processes often result in changes in plasma drug concentrations, which may subsequently influence the anticonvulsant and toxic properties of these drugs. Another consequence of prolonged drug treatment is the development of various adaptive responses, including alterations in receptor sensitivity or density and modifications in gene expression [17,18,19].

It is important to note that the majority of animal studies evaluate the anticonvulsant activity of AEDs only after a single administration of a given anticonvulsant. However, epilepsy requires chronic, even lifelong, treatment. It thus appears reasonable to undertake a detailed comparison between the acute and chronic effects of antiepileptics in the principal screening tests. However, the use of AEDs in chronic treatment significantly increases the number of animals used in experiments. This is unadvantageous from an ethical standpoint.

This comprehensive study is the first to evaluate the impact of the single and repeated administration of VPA on its anticonvulsant efficacy, neurological adverse effects, protective index, as well as the plasma and the brain concentrations in mice. Moreover, the alteration in the expression level of selected genes in the mouse hippocampus was evaluated, following acute and chronic treatment with VPA, in order to provide a molecular basis for the observed results. The following genes were selected for analysis as being responsible for neuronal excitation and inhibition: CACNA1G (encoding subunit of the T-type calcium channel), GABRA1 (encoding subunit of GABA A receptor), GAD1 (encoding glutamate decarboxylase-67), GRIN2B (encoding subunit of the NMDA receptor), and SCN1A (encoding subunit of the sodium voltage-gated channel) [20].

## 2. Results

### 2.1. Behavioral Research

#### 2.1.1. Maximal Electroshock

**Figure 1:**
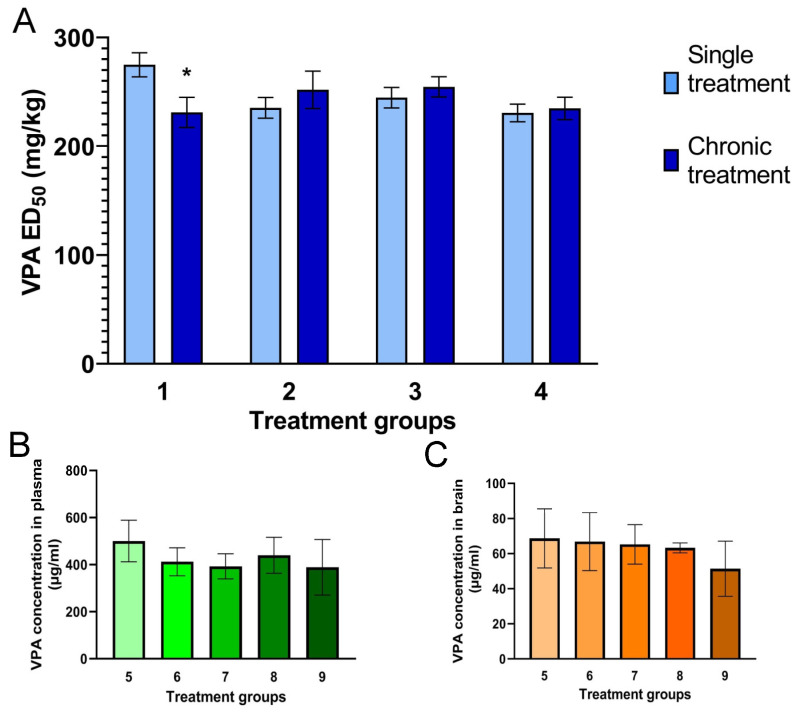
Effect of acute and chronic valproate (VPA) treatment in mice—(**A**) impact of acute and chronic treatment with valproate (VPA) on its anticonvulsant action against maximal electroshock-induced seizures in mice (data are presented as median effective doses (ED_50_ with SEM values) protecting 50% of the animals against seizures. VPA was administered acutely or chronically 30 min before test; treatment groups: 1—2 injections daily for 14 days; 2—1 injection daily for 14 days; 3—2 injections daily for 7 days; 4—1 injection daily for 7 days; * *p* < 0.05 vs. control—single administration of VPA), (**B**) the plasma and (**C**) the brain concentrations of VPA after acute and chronic treatment (results are presented as the means ± SD of at least eight determinations. Statistical analysis of the plasma and the brain concentrations of valproate was performed using one-way analysis of variance (ANOVA) followed by the post hoc Dunnett’s test. Single administration of valproate served as a control (group 5). VPA—valproate; treatment groups: 6—1 injection daily for 7 days; 7—2 injections daily for 7 days; 8—1 injection daily for 14 days; 9—2 injections daily for 14 days.

The antielectroshock activity of VPA altered depending on the regimen of chronic treatment. The ED_50_ (50% effective dose) of VPA given in the 14 × 2 protocol was significantly lower than the control value (1 × 1 protocol): 231.1 ± 13.84 mg/kg (dose B) vs. 274.8 ± 11.07 mg/kg (dose A) [t(62) = 2.466; *p* = 0.0164]. The two doses of VPA were applied in subsequent investigations (animal groups A-1, A-2, B-1, B-2). No significant differences were found in the three remaining drug administration protocols. To exclude the influence of seasonal rhythms on the ED_50_ value [21], each chronic protocol was followed by a new control. The effects of the acute and chronic administration of VPA on maximal electroshock seizures are presented in Figure 1A (see Table S1 and Figure S1 for the details).

#### 2.1.2. Chimney Test

VPA administered acutely or chronically twice a day for 14 days at doses of 274.8 mg/kg (groups A-1 and A-2) and 231.1 mg/kg (groups B-1 and B-2) did not affect motor coordination. All animals left the chimney tube within several seconds (Table 1 and Table S2).

The TD_50_ values after acute and chronic administration (14 × 2) of VPA were determined in the chimney test. The median toxic dose of VPA after a single injection was 465.0 ± 7.45 mg/kg. In the chronic protocol, VPA toxicity was intensified, since TD_50_ (364.1 ± 12.94 mg/kg) was significantly lower compared to the acute experiment [t(130) = 4.817, *p* < 0.0001] (Table 2). Consequently, the protective index of chronic VPA (1.58) was lower than that of the acute VPA (1.69) (Table 2).

#### 2.1.3. Step-Through Passive Avoidance Test

The administration of VPA at a dose of 274.8 mg/kg (group A-1) resulted in a significant impairment in long-term memory as evaluated in the step-through passive avoidance task in mice. No significant deficit in memory was observed after repeated administration (14 × 2) of the same dose of VPA (group A-2) or the lower dose of the antiepileptic (group B-1 and B-2) in the acute and chronic protocols (Table 3 and Table S2).

#### 2.1.4. Brain and Plasma Concentration of VPA

The plasma and the brain concentrations of VPA were not altered during chronic treatment with this drug. The plasma and the brain levels of VPA administered twice daily for 14 days were lower than the plasma and brain levels of acute VPA but the difference did not reach the level of significance (Figure 1B,C and Table S3).

### 2.2. The Assessment of the Expression Level of Selected Genes in the Mouse Hippocampus

#### 2.2.1. Comparison of the Expression Level of Selected Genes in the Hippocampus of Mice After Single and Chronic Treatment with VPA Versus the MW Group (A-1, A-2, B-1, B-2 vs. MW—Calibrator, Control)

**Figure 2:**
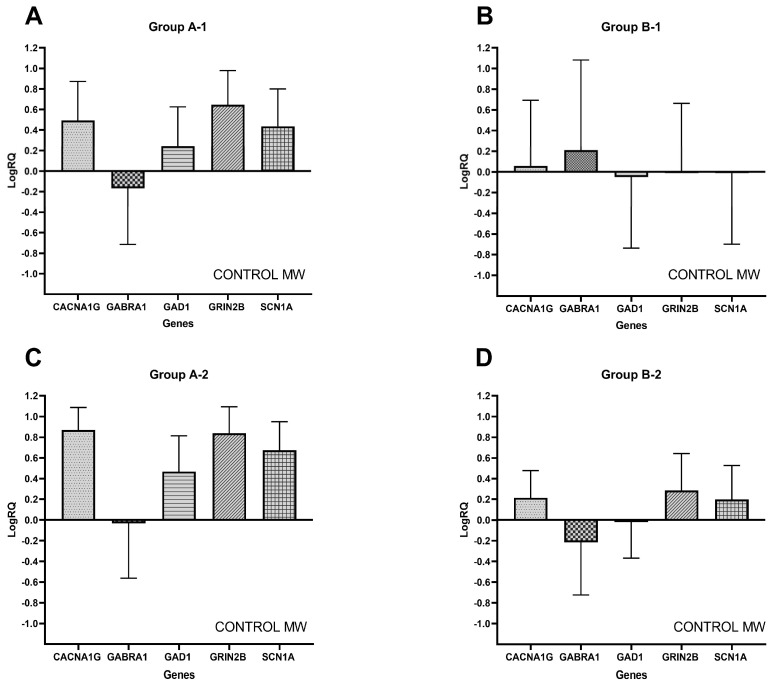
Mean expression profile of the studied genes in the hippocampus of mice treated with VPA in both single (A-1, B-1) and chronic (A-2, B-2) treatment protocols. Data are presented as the mean gene expression levels (on a logarithmic scale LogRQ). Marked SD, standard deviation. (**A**) group A-1, (**B**) group B-1, (**C**) group A-2, (**D**) group B-2. MV—calibrator, control.

The gene expression profile for five selected genes in the mouse hippocampus was studied after single (A-1, B-1) and chronic treatment (A-2, B-2) with VPA administered at two doses and compared to the control animals (MW), which received the vehicle (Figure 2A–D, Table S4). VPA at the higher dose (dose A) increased the expression level of four out of the five tested genes in both protocols (A-1 and A-2). The results of the treatment with VPA at the other dose (B-1 and B-2) were more mixed.

#### 2.2.2. CACNA1G Gene

**Figure 3:**
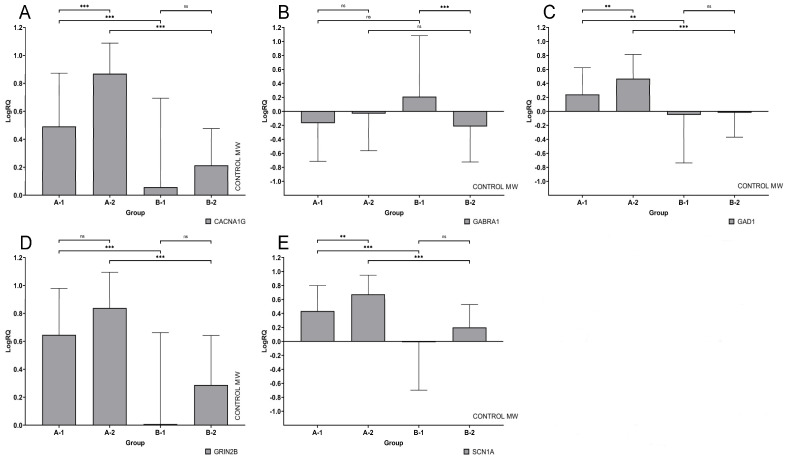
Comparison of the expression level of the studied genes in the mouse hippocampus in both single (A-1, B-1) and chronic (A-2, B-2) treatment protocols with VPA. (**A**) CACNA1G gene, (**B**) GABRA1 gene, (**C**) GAD1 gene, (**D**) GRIN2B gene, (**E**) SCN1A gene. Data are presented as the mean gene expression levels of the studied genes (on a logarithmic scale LogRQ). Marked SD, standard deviation. Indicated statistically significant differences in levels of gene expression between treatment protocols and doses of VPA (Kruskal–Wallis test), ns not statistically significant, ** *p* < 0.01, *** *p* < 0.001. Results after Bonferroni correction.

A single injection of VPA at the higher dose (dose A) increased the CACNA1G gene expression (mean level LogRQ = 0.49; SD = 0.38) in the mouse hippocampus. The chronic treatment resulted in a further increase in the CACNA1G gene expression level (mean level LogRQ = 0.87; SD = 0.22) and the up growth between protocols (groups A-1 and A-2) was statistically significant (*p* < 0.001). A similar effect was observed after treatment with VPA at dose B (mean level LogRQ = 0.06; SD = 0.64 and LogRQ = 0.21; SD = 0.26, respectively); however, it was less pronounced and did not reach statistical relevance. Substantial differences in the gene expression level evoked by the administration of VPA at doses A and B in both protocols were also noted (Figure 3A, Table S5).

#### 2.2.3. GABRA1 Gene

Both the single and chronic administration of VPA at dose A decreased the GABRA1 gene expression level (mean level LogRQ = −0.17; SD = 0.55 and LogRQ = −0.03; SD = 0.53, respectively); there were no statistical differences between groups. A single injection of VPA at dose B increased the GABRA1 gene expression level (mean level LogRQ = 0.21; SD = 0.87) in the mouse hippocampus. The chronic treatment resulted in a reduction in the GABRA1 gene expression level (mean level LogRQ = −0.22; SD = 0.51) and the difference between protocols (groups B-1 and B-2) was statistically significant (*p* < 0.001). No significant differences in gene expression levels induced by the administration of VPA at doses A and B were observed in either protocol (Figure 3B, Table S5).

#### 2.2.4. GAD1 Gene

A single injection of VPA at dose A increased the GAD1 gene expression level (mean level LogRQ = 0.24; SD = 0.38) in the mouse hippocampus. The chronic treatment resulted in a further increase in the GAD1 gene expression (mean level LogRQ = 0.47; SD = 0.34) and the up growth between protocols (groups A-1 and A-2) was statistically significant (*p* < 0.01). Both the single and chronic administration of VPA at dose B decreased the GAD1 gene expression level (mean level LogRQ = −0.05; SD = 0.69 and LogRQ = −0.02; SD = 0.35, respectively); there were no statistical differences between groups. Substantial differences in the gene expression level evoked by the administration of VPA at doses A and B in both protocols were also observed (Figure 3C, Table S5).

#### 2.2.5. GRIN2B Gene

A single injection of VPA at dose A increased the GRIN2B gene expression level (mean level LogRQ = 0.65; SD = 0.33) in the mouse hippocampus. The chronic treatment resulted in a further increase in the GRIN2B gene expression level (mean level LogRQ = 0.84; SD = 0.26) and the up growth between protocols (groups A-1 and A-2) was not statistically significant. The difference between the acute and chronic treatment with VPA at dose B was not statistically significant; however, the mean values of the GRIN2B gene expression level (mean level LogRQ = 0.01; SD = 0.65 and LogRQ = 0.29; SD = 0.35, respectively) were lower. Substantial differences in the gene expression level evoked by the administration of VPA at doses A and B in both protocols were observed (Figure 3D, Table S5).

#### 2.2.6. SCN1A Gene

A single injection of VPA at dose A increased the SCN1A gene expression level (mean level LogRQ = 0.44; SD = 0.36) in the mouse hippocampus. The chronic treatment resulted in a further increase in the SCN1A gene expression level (mean level LogRQ = 0.67; SD = 0.27) and the up growth between protocols (groups A-1 and A-2) was statistically significant (*p* < 0.01). The difference between the acute and chronic treatment with VPA at dose B (mean level LogRQ = −0.01; SD = 0.69 and LogRQ = 0.20; SD = 0.33, respectively) was not statistically significant. Substantial differences in the gene expression level evoked by the administration of VPA at doses A and B in both protocols were noted (Figure 3E, Table S5).

#### 2.2.7. Cluster Analysis and Comparison of the Correlation Between the Expression Level of the Studied Genes

**Figure 4:**
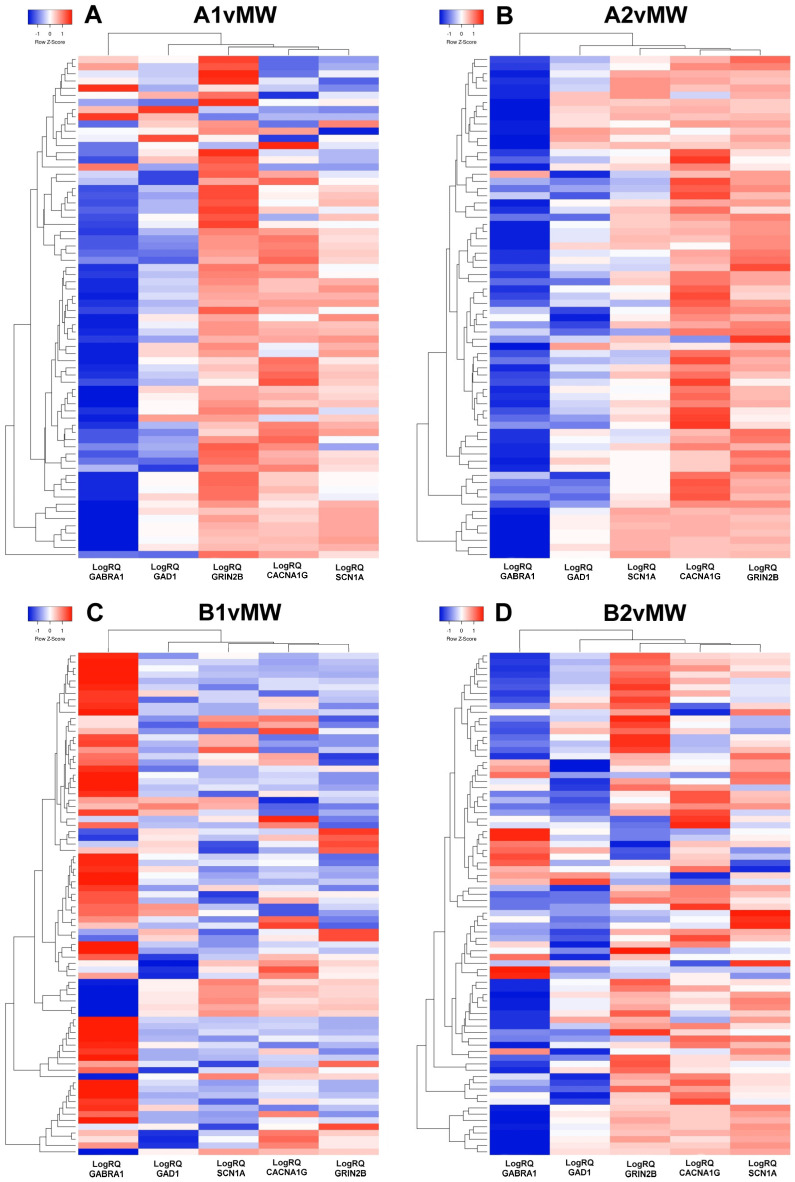
Heatmap of the expression level of the studied genes in the mouse hippocampus in both single (A-1, B-1) and chronic (A-2, B-2) treatment protocols with VPA (relative to MW control). (**A**) A-1 vs. MW, (**B**) A-2 vs. MW, (**C**) B-1 vs. MW, (**D**) B-2 vs. MW.

Heatmaps generated using Heatmapper show the cluster analysis of the expression levels of the studied genes in the mouse hippocampus in both the single (A-1, B-1) and chronic (A-2, B-2) VPA treatment protocols relative to the MW control (Figure 4A–D). The cluster analysis presented as heatmaps in the group of mice after single (A-1) and chronic (A-2) treatment with VPA at dose A is similar (relative to MW control). The heatmaps show the separation of the reduced expression level of the GABRA1 gene from the group of other genes that were characterized by a more diverse expression level. A similar cluster analysis performed in a group of mice after single (B-1) treatment with VPA at dose B (relative to MW control) allowed for the separation of an increased expression level of the GABRA1 gene from the group of other genes that were characterized by a more diverse expression level. Meanwhile, cluster analysis in a group of mice after (B-2) chronic treatment with VPA at dose B (relative to MW control) isolated the reduced expression level of the GABRA1 gene from the group of other genes that were characterized by a more diverse expression level.

**Figure 5:**
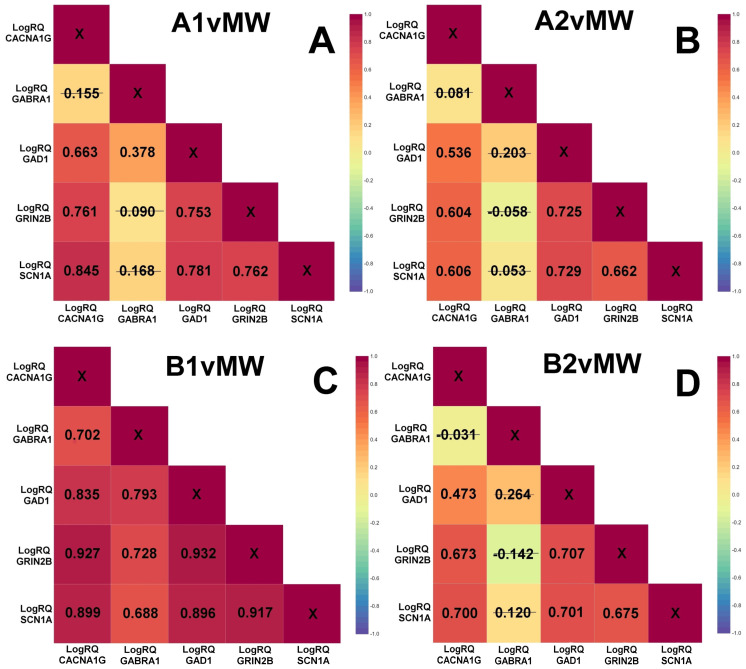
Correlation between the expression level of the studied genes in the mouse hippocampus in both single (A-1, B-1) and chronic (A-2, B-2) treatment protocols with VPA (relative to MW control). (**A**) A-1 vs. MW, (**B**) A-2 vs. MW, (**C**) B-1 vs. MW, (**D**) B-2 vs. MW (*p* < 0.05). Values that are not statistically significant are crossed out. Results after Bonferroni correction.

Correlation analysis (r-Spearman) showed that all statistically significant correlations between the studied genes were positive (Figure 5, Table S6). Gene correlations in the group of mice after single (A-1) and chronic (A-2) treatment with VPA at dose A are similar. The highest correlation coefficients are between the CACNA1G gene and SCN1A, GRIN2B, and GAD1 genes. A strong correlation was also observed between GAD1 and SCN1A and GRIN2B and between GRIN2B and SCN1A. Gene correlation analysis in a group of mice after single (B-1) treatment with VPA at dose B showed a high or very high correlation between all studied genes. Meanwhile, correlation analysis of genes in a group of mice after chronic (B-2) treatment with VPA at dose B showed a correlation between the CACNA1G gene and SCN1A, GRIN2B, and GAD1 genes, as well as between GAD1 and SCN1A and between GRIN2B and SCN1A. Statistically insignificant relationships were removed from the correlation heatmaps.

#### 2.2.8. Comparison of the Expression Level of Selected Genes in the Hippocampus of Mice After Chronic Treatment with VPA Versus Single Treatment with VPA (A-2 vs. A-1—Calibrator, Control and B-2 vs. B-1—Calibrator, Control)

**Figure 6:**
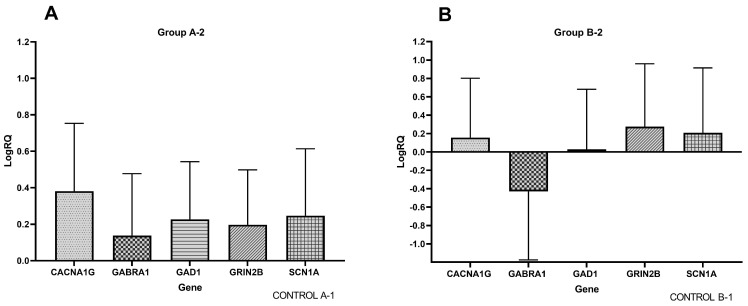
Mean expression profile of the studied genes in the hippocampus of mice treated with VPA in chronic treatment protocols (A-2, B-2). Data are presented as the mean gene expression levels (on a logarithmic scale LogRQ). Marked SD, standard deviation. (**A**) group A-2, (**B**) group B-2.

The gene expression profile for five selected genes in the mouse hippocampus was studied after chronic treatment with VPA administered at two doses (groups A-2, B-2) and compared to the control animals (groups A-1, B-1), which received single treatment with VPA (Figure 6, Table S7). VPA at dose A (the higher one, group A-2) increased the expression level of all tested genes. The results of the treatment with VPA at dose B (the lower one, group B-2) increased the expression level of the CACNA1G, GAD1, GRIN2B and SCN1A genes, while decreasing the expression level of the GABRA1 gene.

**Figure 7:**
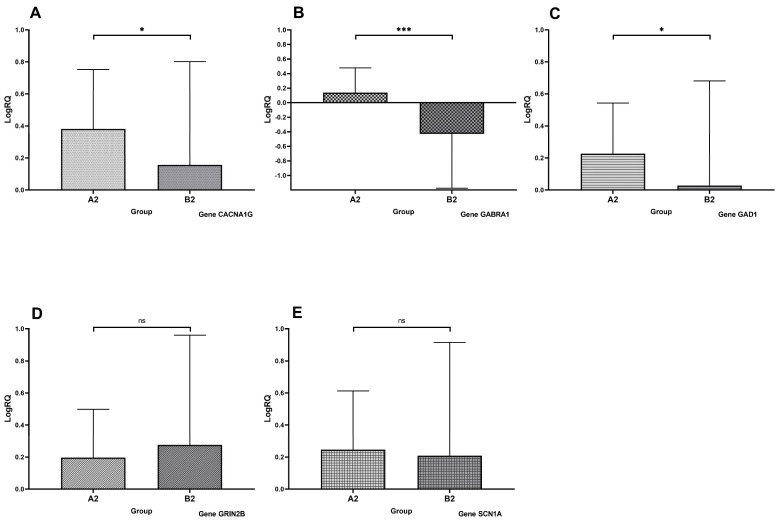
Comparison of the expression level of the studied genes in the mouse hippocampus in chronic (A-2, B-2) treatment protocols with VPA (vs. single treatment protocols with VPA—A-1, B-1, respectively). (**A**) CACNA1G gene, (**B**) GABRA1 gene, (**C**) GAD1 gene, (**D**) GRIN2B gene, (**E**) SCN1A gene. Data are presented as the mean gene expression levels of the studied genes (on a logarithmic scale LogRQ). Marked SD, standard deviation. Indicated statistically significant differences in levels of gene expression between treatment protocols of VPA (U Mann–Whitney test), ns—not statistically significant,* *p* < 0.05, *** *p* < 0.001.

A comparative analysis of the expression level of selected genes in the mouse hippocampus after chronic treatment versus single treatment with VPA at the two doses (A-2 vs. A-1—calibrator, control and B-2 vs. B-1—calibrator, control) was also performed (Figure 7, Table S8). Chronic administration of VPA at dose A (A-2) increased the expression levels of the all tested genes: CACNA1G (mean level: LogRQ = −0.38; SD = 0.37), GABRA1 (mean level: LogRQ = −0.14; SD = 0.34), GAD1 (mean level: LogRQ = −0.23; SD = 0.32), GRIN2B (mean level: LogRQ = −0.20; SD = 0.30) and SCN1A (mean level: LogRQ = −0.25; SD = 0.37). However, chronic injection of VPA at dose B decreased the GABRA1 gene expression level (mean level LogRQ = −0.43; SD = 0.74) and increased the expression levels of the four remaining genes: CACNA1G (mean level: LogRQ = −0.16; SD = 0.65), GAD1 (mean level: LogRQ = −0.028; SD = 0.65), GRIN2B (mean level: LogRQ = −0.28; SD = 0.68) and SCN1A (mean level: LogRQ = −0.21; SD = 0.71). Statistically significant differences were observed in the expression levels of the GABRA1 (*p* < 0.001), CACNA1G (*p* < 0.05) and GAD1 (*p* < 0.05) genes between groups A-2 and B-2.

**Figure 8:**
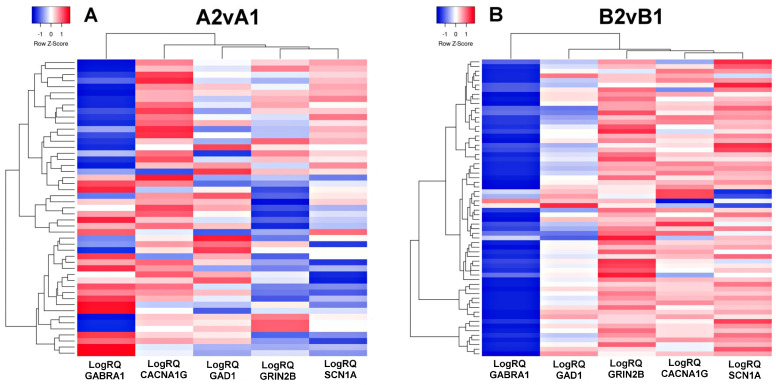
Heatmap of the expression level of the studied genes in the mouse hippocampus in chronic treatment protocols (A-2, B-2) with VPA (relative to A-1 and B-1 control, respectively). (**A**) A-2 vs. A-1, (**B**) B-2 vs. B1.

Cluster analysis presented in the form of heatmaps in the group of mice (A-2) after chronic treatment with VPA at dose A distinguished a differentiated level of GABRA1 gene expression from the group of other genes. Meanwhile, in the group of mice (B-2) after chronic treatment with VPA at dose B, a reduced level of GABRA1 gene expression was identified from a group of other genes that were characterized by a more diverse expression level (Figure 8).

**Figure 9:**
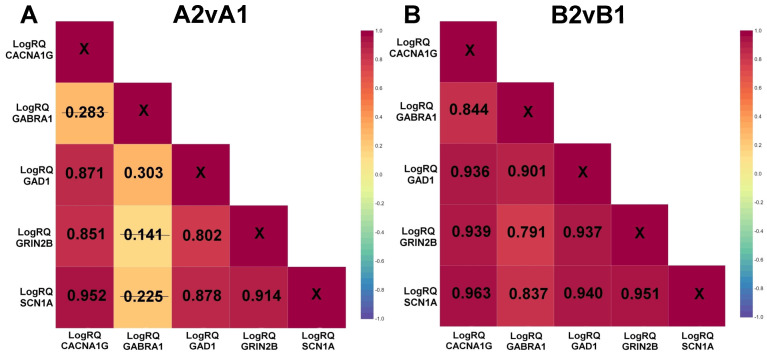
Correlation between the expression level of the studied genes in the mouse hippocampus in chronic (A-2, B-2) treatment protocols with VPA (relative to A-1 and B-1control, respectively). (**A**) A-2 vs. A-1, (**B**) B-2 vs. B1 (*p* < 0.05). Values that are not statistically significant are crossed out. Results after Bonferroni correction.

Gene correlations (r-Spearman) in the group of mice after chronic (A-2) treatment with VPA at dose A were positive. The highest correlation coefficients are between the CACNA1G gene and SCN1A, GRIN2B, and GAD1 genes. A strong correlation was also observed between GAD1 and SCN1A and GRIN2B and between GRIN2B and SCN1A. Meanwhile, correlation analysis in a group of mice after chronic (B-2) treatment with VPA at dose B showed a high or very high correlation between all studied genes. Statistically insignificant relationships were removed from the correlation heatmaps (Figure 9, Table S9).

#### 2.2.9. Functional Analysis of the Studied Genes

**Figure 10:**
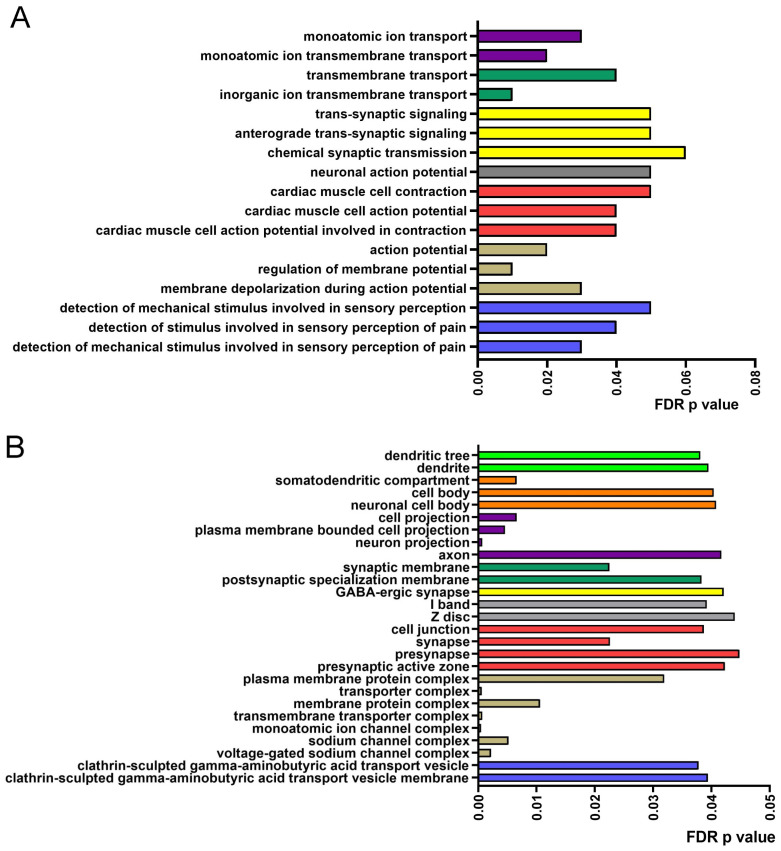

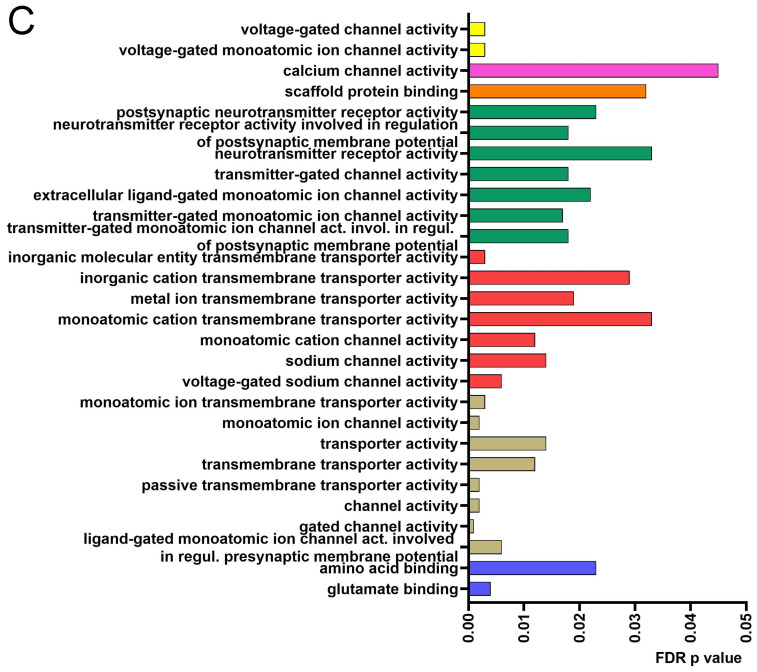
Functional analysis of the studied genes carried out using the PANTHER v.18.0 classification system—(**A**–**C**) GO biological process complete, GO cellular component complete, GO molecular function complete.

During the study, a preliminary functional analysis of the genes under investigation was also performed. The software used enables the automatic evaluation and classification of the results as statistically significant or not significant. Only those results identified by the program as statistically significant were included in further analyses; however, it should be emphasized that due to the small number of analyzed genes, the obtained results are primarily illustrative and represent a form of targeted exploratory analysis requiring further validation in subsequent studies. The functional analysis was conducted in three categories: GO Biological Process, GO Cellular Component, and GO Molecular Function. The detailed results of the functional analysis are presented in Figure 10.

## 3. Discussion

Epilepsy is a prevalent chronic neurological disorder that necessitates long-term pharmacotherapy. However, even rational polytherapy has no significant effect in approximately 30% of patients. One hypothesis suggests that the phenomenon of drug resistance may have a genetic basis. Single AED administration, preferred in the majority of experimental studies, does not take into account the impact of the potential pharmacological and epigenetic changes induced by chronic therapy. In light of this, a comparative study was conducted to investigate the impact of single and chronic treatment with VPA in mice on behavioral outcomes (anticonvulsant efficacy, neurological adverse effects, and the protective index), the plasma and brain concentrations of the drug, and alterations in the expression of selected genes in the hippocampus.

The present study demonstrated that the anticonvulsant action of VPA in mice is amplified when administered twice daily for a period of 14 days, a finding that was replicated in the case of lamotrigine [22]. However, the interpretation of this phenomenon remains unsupported by the extant scientific literature. The extant data regarding the chronic administration of VPA are limited, contradictory, and contingent upon the specific seizure model, dosage, and treatment protocol [11] (Table 4). The results of the present study can be considered to be supported by Löscher and Schmidt [23] in kindled rats given a 7-day treatment with primidone or vigabatrin. This finding corroborates the hypothesis that a single administration of AEDs may result in an underestimation of their anticonvulsant properties. However, in contrast to the observations reported herein, the antiseizure action of the two AEDs was enhanced mainly due to pharmacokinetic reasons.

In a clinical setting, a fully developed anticonvulsant effect of VPA is usually observed after 1–2 weeks of regular use. The efficacy of VPA varies by patient and seizure type. A 3-year VPA monotherapy trial in 115 patients with primary generalized seizures achieved complete control in 74% of cases, with 9% experiencing occasional seizures [41]. In a subsequent study of 24 patients with refractory complex partial seizures, 50% exhibited a substantial reduction in seizures, while only 21% demonstrated long-term improvement, and 29% exhibited drug tolerance [42]. Another study, which examined 34 children with various seizure types, reported an 82% success rate in achieving complete control, with 18% experiencing recurrence [43]. A notable limitation of these studies is the absence of assessment of VPA effectiveness at varying stages of treatment.

In the present study, the administration of VPA, either as an acute or chronic treatment, did not result in alterations in mouse motor performance. However, it was observed that the 50% toxic dose was reduced following chronic treatment in comparison to a single injection. Despite this, the protective index remained unaltered due to a concurrent decrease in the 50% effective dose. While VPA did not affect motor function, a single dose, but not chronic treatment, significantly impaired long-term memory. This suggests that chronic therapy may exacerbate motor deficits but could also indicate tolerance to memory impairment. Consistent with our findings, chronic administration of lamotrigine increased motor deficits compared to acute treatment [22].

The effects of chronic VPA administration on animal memory are inconsistent, varying with seizure models and memory tests. In pentylenetetrazole-kindled mice, chronic VPA (300 mg/kg/24 h *i.p.* for 20 days) reduced memory deficits, likely due to increased dopamine and nitrite levels and decreased acetylcholinesterase activity in the hippocampus [27]. VPA improved recognition memory and spatial learning in Alzheimer’s disease transgenic mice [44]. However, a 10-day VPA treatment (300 mg/kg i.p.) impaired spatial memory in rats [45], and chronic VPA (400 mg/kg *p.o.*) did not improve non-spatial memory in transgenic BSN mouse mutants [36]. In contrast, chronic oral VPA (370 mg/kg for 45 days) did not impair memory in various tests in rats [46]. In epilepsy patients, VPA is considered a safer option for cognitive functions compared to other AEDs, though cognitive decline may occur as epilepsy progresses [47,48,49,50].

It is plausible that the anticonvulsant and adverse effects of VPA may share underlying mechanisms, although direct pharmacokinetic mechanisms have been excluded. One important limitation of our study is the fact that a fluorescence polarization immunoassay may cross-react with VPA metabolites such as 4-ene-VPA and 3-OH-VPA, so we cannot exclude shifts in metabolite profiles. The influence of VPA metabolites on its pharmacological profile is a potential area of investigation. However, the hypothesis that metabolite accumulation may enhance VPA’s antiseizure efficacy after chronic treatment has not been substantiated by evidence [24,28,29]. Noteworthy, rodent pharmacokinetics differ from those of humans. A detailed analysis of the therapeutic and toxic effects of VPA metabolites in humans was conducted by Shnayder et al. [51].

In consideration of the previously mentioned data, the augmented antielectroshock effect of chronic VPA therapy may be attributable primarily to pharmacodynamic mechanisms and accompanied by transcriptional adaptations induced by prolonged drug exposure. VPA has been shown to block voltage-gated sodium and calcium (T, N, and L) channels [26,52,53], increase GABA synthesis [54,55], decrease its degradation, and modulate gene expression [53]. A 12-day administration of VPA (500–580 mg/kg *p.o.*) has been reported to increase brain GABA levels through enhanced GAD activity [25] and to reduce glutamate, asparagine, taurine, and alanine concentrations [54,55]. Similar alterations were observed in the plasma and cerebrospinal fluid of patients with epilepsy [54]. Moreover, VPA administered at 200 mg/kg/day i.p. for 30 days inhibited dopaminergic and glutamatergic neurotransmission [14,15].

Although VPA is recognized as a histone deacetylase inhibitor and has been associated with the modulation of gene expression through DNA and histone-related mechanisms, the present study assessed only mRNA expression levels [26,44]. Therefore, the observed transcriptional changes should be interpreted cautiously and cannot be considered direct evidence of epigenetic regulation. These alterations may also result from other regulatory processes, including changes in transcription factor activity or mRNA stability. Further studies involving direct epigenetic analyses, such as chromatin immunoprecipitation, histone modification assays, DNA methylation analysis, and protein expression assessment, are required to clarify the molecular mechanisms underlying the effects of chronic VPA treatment.

However, irrespective of the precise mechanism of action, VPA has been demonstrated to promote chromatin decondensation, thereby facilitating the expression of numerous genes [56]. According to Fukuchi et al. [57], this AED increased the expression of 726 genes while decreasing the expression of 577 genes in cultured rat cortical neurons. Furthermore, a study by Bosetti et al. [58] found that i.p. injection of VPA (200 mg/kg) over 30 days led to the upregulation of 34 genes and downregulation of 87 genes in the rat brain. Among the genes that were found to be upregulated, there were some linked to epileptogenesis, including those encoding the brain-derived neurotrophic factor and the alpha4 subunit of the GABAA receptor [57]. In epilepsy patients, a 3-month treatment period with therapeutic doses of VPA resulted in significant differences in the expression of 11 out of 23,099 genes analyzed in the blood, including those related to cancers and mitochondrial function [57]. Among AEDs, VPA is the most potent inducer of mitochondrial toxicity [59].

The present study investigated the impact of chronic VPA treatment on the expression levels of five genes in the mouse hippocampus (CACNA1G, GABRA1, GAD1, GRIN2B, and SCN1A) that play a role in neuronal excitability [60,61]. 

The analysis revealed that a lower VPA dose (231.1 mg/kg) led to a significant decrease in GABRA1 expression, while the expression of the other genes remained relatively unchanged. Conversely, a higher VPA dose (274.8 mg/kg) elicited a substantial increase in CACNA1G, GAD1 and SCN1A expression, accompanied by a marginal rise in GABRA1 expression. These observations offer a potential explanation for the augmented efficacy and toxicity associated with chronic VPA administration.

Voltage-gated T-type calcium channels have been identified as playing an important role in the pathogenesis of seizures. CACNA1G, which encodes the alpha1G/Cav3.1 subunit of this channel, has been identified as a genetic modifier of epilepsy. In experimental models of pure absence epilepsy, CACNA1G over-expression in C57BL/6, tottering, lethargic, stargazer, and coloboma mice has been demonstrated to exacerbate seizure severity [62]. In contrast, mice lacking the α1G subunit of T-type calcium channels exhibited resistance to absence seizures induced by GABAB receptor activation [63]. Furthermore, CACNA1G over-expression in the mouse SCN2A transgenic epilepsy model of focal motor seizures (with a gain-of-function mutation) resulted in an elevated frequency of focal seizures. Conversely, decreased expression of CACNA1G reduced spontaneous seizures in a mouse model of the SCN1A^+/−^ Dravet phenotype, but increased expression of this gene had no effect on seizure parameters [64]. These findings suggest that modulation of CACNA1G may be an effective therapeutic strategy in selected patients. In vitro, CACNA1G expression increased after 14 days of exposure of human mesenchymal stem cells to VPA (10 µM) [65]. This finding was confirmed in our study. Chronic treatment with the higher dose of VPA resulted in a significant increase in the expression of CACNA1G. However, T-type calcium channels play an essential role in the propagation of absence seizures. Our study was conducted using a model of generalized tonic–clonic convulsions, in which the role of T-type channels is very limited. Therefore, to explain the functional consequences of CACNA1G transcriptional changes, direct electrophysiological or protein-level validation is required.

GABAA receptors belong to ligand-gated chloride channels that play a crucial role in mediating inhibitory postsynaptic currents. The GABRA1 gene, which is responsible for encoding the alpha 1 subunit, has been associated with absence, febrile, and generalized seizures [20,66]. Conversely, Kumari et al. [67] observed an elevated risk of multidrug-resistant epilepsy in the North Indian population associated with GABRA1 variants. However, a subsequent meta-analysis by Zhang et al. [68] failed to establish a link between the GABRA1 (rs2279020) polymorphism and refractory epilepsy in Asian and Arabic patients. Prenatal VPA exposure has been shown to reduce GABRA1 mRNA and protein levels in the mouse cortex [69]. In rat cortical neurons, VPA has been observed to increase mRNA for the GABAARα4 and γ1 subunits, involved in epileptogenesis, while reducing the GABAARγ2 subunit, which is linked to inhibitory neuron development. These effects were replicated with trichostatin A, suggesting that VPA exerts its effects by inhibiting histone deacetylases [57]. The present study demonstrated that chronic low-dose VPA led to a significant decrease in GABRA1 expression, while high-dose VPA resulted in an insignificant increase.

The GAD1 and GAD2 genes encode GAD67 and GAD65, enzymes that convert glutamate to GABA [70]. GAD67 is responsible for basal GABA synthesis, while GAD65 is activated under conditions of increased GABA demand. In humans and rats, GAD65 expression exceeds GAD67, while in mice, their expression is comparable [71,72]. GAD67-deficient mice manifest severe developmental abnormalities and reduced cortical GAD and GABA levels, while GAD65-deficient mice exhibit spontaneous seizures [71,73,74,75]. In humans, bi-allelic GAD1 mutations have been associated with seizures, hypotonia, and developmental delay [72]. VPA has been observed to influence GAD1 expression by modulating histone acetylation and DNA methylation [70]. In mice, the administration of VPA (300 mg/kg/day for 7 days) has been shown to enhance GABAergic transmission by reducing GAD1 promoter methylation [73]. However, in rat cortical neurons, VPA and trichostatin A have been observed to reduce GAD67 mRNA and protein expression [57]. Prenatal VPA exposure has decreased GAD67 protein but not mRNA levels, suggesting a post-transcriptional effect [69]. The present study found that chronic high-dose VPA significantly increased GAD1 expression in the mouse hippocampus, while low-dose VPA showed a slight, insignificant decrease. Increased GAD1 mRNA has been linked to enhanced GABA release from NPY/AgRP neurons [76].

The GRIN2B gene, which is responsible for encoding the glutamate-binding subunit of the NMDA receptor, is essential for synaptic transmission, neuronal plasticity, and excitotoxicity [77,78]. A growing body of research has identified a correlation between gain-of-function and loss-of-function GRIN2B mutations and epileptic syndromes, including West syndrome, childhood-onset focal epilepsy, and neurodevelopmental disorders [66,79,80]. In rats with kainate-induced status epilepticus, reduced GRIN2B mRNA and protein expression in the hippocampus was observed [81]. On the other hand, enhanced spatial memory and increased hippocampal long-term potentiation were observed in mice with the over-expression of GRIN2B in the forebrain [80]. The present study found that chronic VPA insignificantly increased GRIN2B expression in the mouse hippocampus. A single VPA dose resulted in impaired long-term memory in the passive avoidance test; however, chronic treatment did not cause memory deficits. Further studies, including direct electrophysiological or protein-level validation, are necessary to elucidate the functional consequences of our results.

Guo et al. [82] observed increased hippocampal SCN1A mRNA and Nav1.1 protein expression in spontaneously epileptic rats, correlating with elevated sodium currents in hippocampal cultures and tissue [83]. In a mouse model of Dravet syndrome, stimulating SCN1A expression using antisense oligonucleotides reduced electrographic seizures and the rate of sudden unexpected death [84]. Dravet syndrome is primarily caused by SCN1A mutations leading to Nav1.1 haploinsufficiency [85]. Our study showed that chronic high-dose VPA treatment significantly increased hippocampal SCN1A gene expression, suggesting a potential mechanism for VPA’s therapeutic effects.

The present study demonstrated that chronic high-dose VPA treatment significantly increased the expression of the CACNA1G, GAD1, and SCN1A genes. The upregulation of SCN1A and GAD1 may contribute to VPA’s antielectroshock effects. Moreover, low-dose VPA treatment resulted in elevated GABRA1 expression, which may potentially diminish the drug’s anticonvulsant effectiveness. The observed gene expression patterns suggest an adaptive up- and downregulation of VPA targets, including CACNA1G, SCN1A, GAD1, and GABRA1. It is noteworthy that the expression of GABRA1 was significantly reduced exclusively by low-dose VPA treatment.

Correlation and functional analysis revealed that all statistically significant correlations between the investigated genes were positive, with most exhibiting high or very high correlation coefficients. This finding suggests that an increase in the expression of one gene is associated with an increase in others, indicating the presence of common regulatory mechanisms and involvement in shared intracellular pathways. Functional analysis confirmed that the tested genes play a role in neurotransmission regulation.

## 4. Materials and Methods

### 4.1. Animals

Experiments in the present study were conducted on adult male Swiss mice weighing 20–26 g. The animals were housed in appropriately sized cages with a floor size of 820 cm^2^ for 8 to 10 mice (W × L × H: 378 × 217 × 180 mm). The animals had free access to food (maintenance diet for mice: Altromin 1324 IRR) and tap water. Standardized laboratory conditions were maintained, including a natural light–dark cycle 12 h per day, a temperature range of 20–24 °C, an air humidity of 45–65%, and an air exchange rate of 15 per hour. After a 7-day acclimatization period, the animals were selected randomly from the cages at the time of allocation to the experimental groups. The experimental groups consisted of 8 (seizure test) or 10 mice (the other procedures). All experiments were performed between 9.00 a.m. and 2.00 p.m., with the exception of drug administration. Intraperitoneal injections were performed between 7.30 a.m. and 8.30 a.m. Additionally, in two injection protocols, the second dose of drug was administered between 7.30 p.m. and 8.30 p.m. Each mouse was utilized only once. In total, 624 mice were utilized throughout the entire study. All the investigations were approved by the Local Ethical Committee (license No 10/2008 No 9/2012 and No 44/2020) and complied with EU Directive 2010/63/EU for animal experimentation, as well as ARRIVE guidelines.

### 4.2. Behavioral Research

#### 4.2.1. Drug

Sodium valproate (VPA, Sigma-Aldrich, St. Louis, MO, USA) was prepared by dissolution in distilled water. Each day, fresh solutions were prepared. The antiepileptic was administered intraperitoneally (i.p.) in a volume of 0.01 mL/g body weight.

The chronic treatment with VPA was conducted in accordance with four protocols:

1. Two injections administered daily, with a 12 h interval, for 14 days (14 × 2 chronic treatment);
2. A single injection administered every 24 h for 14 days (14 × 1 chronic treatment);
3. Two injections administered daily, with a 12 h interval, for 7 days (7 × 2 subchronic treatment);
4. A single injection administered every 24 h for 7 days (7 × 1 subchronic treatment).

In the single treatment protocols (1 × 1), serving as a control, animals received injections with the vehicle according to the above schedules and single injection of VPA on the last day of the experiment. In both the acute and chronic tests, VPA was administered 30 min prior to the commencement of experiments.

#### 4.2.2. Maximal Electroshock Seizure Test

The anticonvulsant effect of VPA was estimated in the maximal electroshock (MES) test in mice. The MES test is a well-established animal model of tonic–clonic seizures commonly utilized in the preclinical evaluation of anticonvulsant properties of the tested molecules [86]. The parameters of the rodent shocker generator (Type 221, Hugo Sachs Elektronik, Freiburg, Germany) and the detailed methodology were previously described by Banach and Borowicz [22].

**Figure 11:**
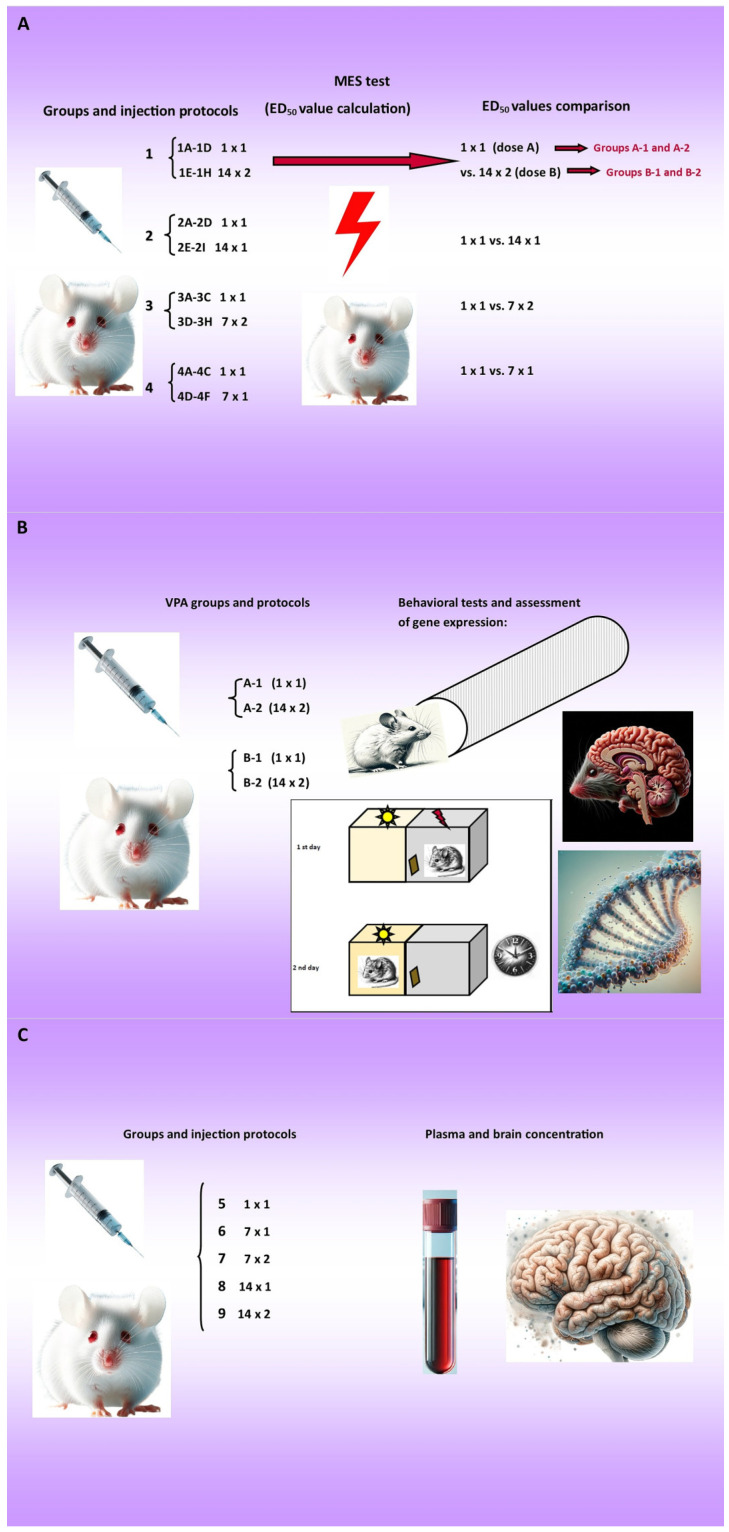
Experimental design and treatment groups. (**A**) Mice were injected with VPA according to four different treatment protocols, then subjected to the MES test. The ED_50_ values were assessed for VPA in every protocol of administration and statistically analyzed. ED_50_—the median effective dose. (**B**) Mice were chronically injected with VPA at the two doses, A and B, then subjected to the behavioral tests (chimney test and passive avoidance task). Corresponding groups of animals were sacrificed for hippocampus isolation and gene expression analysis. (**C**) Mice were injected with VPA according to four chronic treatment protocols. Samples of blood and brains were used to measure plasma and brain concentrations of VPA. VPA—valproate. The graphical content of this illustration was prepared using Microsoft 365 Word and Copilot Version 30.0, (Microsoft® Word and Publisher for Microsoft 365 MSO (version 2605 build 16.0.20026.20140) 64-bit) which is based on artificial intelligence (AI) technology.

The antielectroshock activity of VPA was expressed as the median effective dose (ED_50_), which represents the ability of the drug to protect 50% of animals against maximal electroshock-induced tonic hindlimb extension. A dose–response curve was calculated on the basis of the percentage of mice protected (protection in less than 50%, around 50% and more than 50% of animals) according to Litchfield and Wilcoxon [87]. The ED_50_s values obtained in the first scheme were designated as dose A (1 × 1 single treatment protocol) and dose B (14 × 2 chronic protocol). The two doses of VPA were applied in subsequent investigations (animal groups A-1, A-2, B-1, B-2). The schematic of the experiments is presented in Figure 11A.

#### 4.2.3. Chimney Test

The effects of acute and chronic treatment with VPA (the protocol providing the maximal total dose of VPA: drug injected twice a day for 14 days) at ED_50_s values (dose A and dose B) on motor performance in mice were determined in the chimney test [88]. The following groups of mice were employed:

MW—Mice that were administered water (aqua pro injectione) for 14 days;

A-1—Mice administered water (aqua pro injectione) for 14 days and VPA administered on day 15 in a single dose of 274.8 mg/kg (dose A);

A-2—Mice administered VPA for 14 days twice a day at the dose of 274.8 mg/kg (dose A), B-1—mice administered water (aqua pro injectione) for 14 days and VPA administered on day 15 in a single dose of 231.1 mg/kg (dose B);

B-2—Mice administered VPA for 14 days twice a day at the dose of 231.1 mg/kg (dose B). The last dose of VPA in the chronic regimen (on day 15) was injected 30 min before the procedure (the same time as scheduled for the electroconvulsive test).

The results are presented as a percentage of animals that were unable to complete the test. The detailed methodology was previously described by Banach and Borowicz [22]. The schematic of the experiments is presented in Figure 11B.

The neurotoxic effect of VPA was expressed as the median toxic dose (TD_50_) in mg/kg, which represents the dose at which VPA evoked motor impairment in the chimney test in 50% of mice. To determine TD_50_, at least four groups of 10 mice were injected with progressively increasing doses of the drug and subsequently challenged with the chimney test. A dose—response curve was subsequently calculated based on the percentage of mice exhibiting motor deficits.

The protective index was calculated by dividing TD_50_ by ED_50_ obtained in the acute and chronic (14 × 2) protocols, as described by Löscher and Nolting [89].

#### 4.2.4. Step-Through Passive Avoidance Test

The step-through passive avoidance test, regarded as a measure of long-term memory, is based on the natural reflex of being in the dark [90]. Mice were treated with a single-dose and a chronic treatment (14 × 2) with VPA at ED_50_s values (groups A-1, A-2, B-1, B-2, corresponding to those used in the chimney test) (Figure 11B). Then, they were placed in an illuminated box (10 × 13 × 15 cm) connected to the larger dark box (25 × 20 × 15 cm) equipped with an electric grid floor. The entrance of the animal to the dark box was punished by an adequate electric foot shock (0.6 mA for 2 s). Mice that did not enter the dark compartment within 60 s were excluded from the further experiment. Twenty-four hours later, the pre-trained mice were placed again into the illuminated box and observed for up to 180 s. Animals that avoided the dark compartment for 180 s were considered to have remembered the task. The control group (vehicle-treated mice) did not enter the dark box within the observation period. The time taken by the mice to enter the dark box was recorded, and the median latencies (retention times) with 25th and 75th percentiles were calculated.

#### 4.2.5. Measurement of Plasma and Brain Concentrations of VPA

The animals were administered VPA according to four distinct chronic protocols (groups 6–9, Figure 11C). A single treatment protocol was used as a control group (group 5). The final dose of VPA in the chronic regimen was administered 30 min prior to the respective procedure, at the same time as scheduled for the electroconvulsive test. The precise methodology was described by Banach and Borowicz [22].

The plasma and the brain concentrations of VPA were determined by a fluorescence polarization immunoassay, using the TDx analyzer and reagents in accordance with the manufacturer’s instructions (Abbott Laboratories, North Chicago, IL, USA). The results were expressed in μg/mL and subsequently computed as means ± SD of at least eight determinations.

### 4.3. The Assessment of the Expression Level of Selected Genes in the Mouse Hippocampus

#### 4.3.1. Material Preparation and Study Groups

The assessment of the expression level of selected genes (CACNA1G, GABRA1, GAD1, GRIN2B, SCN1A) in the mouse hippocampus was carried out in both the single and chronic VPA treatment protocols. The experimental groups corresponded to those used in the chimney test and passive avoidance task, that is, A-1, A-2, B-1, B-2 and MW. Mice were killed by decapitation. Isolation of the adult mouse hippocampus was performed according to the instruction provided by the European Journal of Neuroscience [91].

#### 4.3.2. Isolation of Genetic Material and Real-Time PCR

mRNA was isolated using a modified Chomczyński and Sacchi method using TriReagent reagent (Applied Biosystems, Foster City, CA, USA) [92]. The isolated material was quantitatively and qualitatively assessed using a NanoDrop ND-2000 spectrophotometer (ThermoFisher Scientific, Waltham, MA, USA) in order to confirm its appropriate purity and suitability for further molecular analyses. Next, cDNA was synthesized using the “High Capacity cDNA Reverse Transcription Kit,”(Applied Biosystems, Foster City, CA, USA)and the resulting cDNA was used for further gene expression analysis. The reverse transcription reaction was carried out in a thermocycler under the following incubation conditions: step I—25 °C for 10 min, step II—37 °C for 120 min, and step III—85 °C for 5 s (Applied Biosystems, Foster City, CA, USA). The cDNA obtained after reverse transcription was amplified according to the previously described procedure, using the relative quantitative PCR using the ΔΔCt method (Applied Biosystems 7900 instrument (Applied Biosystems, Foster City, CA, USA) and SDS (version 2.4.1, Applied Biosystems, Foster City, CA, USA) and ExpressionSuite Software version 1.3 (ThermoFisher Scientific, Waltham, MA, USA)). The reaction mixture per single reaction contained: 1.25 μL of TaqMan Gene Expression Assays probe together with complementary primers (Applied Biosystems, Foster City, CA, USA), 12.5 μL of TaqMan™ Gene Expression Master Mix buffer (Applied Biosystems, Foster City, CA, USA), and 11.25 μL of the tested cDNA (cDNA diluted in DNase-, RNase-, and protease-free water) [93,94].

Assessment of the impact of VPA administration on the expression level of selected genes in the mouse brain was performed in 96-well plates. TaqMan probes (Applied Biosystems, Foster City, CA, USA) were used for the study of CACNA1G (Mm00486572_m1), GABRA1 (Mm00439046_m1), GAD1 (Mm00725661_s1), GRIN2B (Mm00433820_m1), SCN1A (Mm00450580_m1) and GAPDH (Mm99999915_g1)—control gene. The real-time PCR reaction conditions were consistent with the previously described procedure. The reaction was performed according to the following thermal cycling program: an initial denaturation at 95 °C for 10 min, followed by 40 amplification cycles consisting of step I—denaturation at 95 °C for 15 s and step II—at 60 °C for 60 s [93]. In order to determine the relative expression level of the studied genes, the comparative method (ΔΔCt) was used [95,96]. The test sample was material collected from mice chronically treated with VPA (A-2 and B-2), while the calibrator was material collected from mice not treated with any drug (MW) and in the second analysis of mice after a single dose of VPA (A-1 and B-1). Calculations were performed in parallel for the test and calibrator samples [95] as follows: ΔCt (test sample) = Ct of the target gene − Ct of the reference gene, ΔCt (calibrator) = Ct of the target gene − Ct of the reference gene. Next, the ΔΔCt value was calculated for each test sample: ΔΔCt = ΔCt (unknown sample) − ΔCt (calibrator). The relative expression level of the tested genes in the test sample was analyzed in relation to the calibrator according to the formula RQ = 2^−ΔΔCt^. To facilitate the interpretation of the results, RQ values were transformed into a logarithmic scale (log10), yielding logRQ values [93,95,96,97]. Gene expression normalization in our study is based on the widely accepted Livak method in molecular biology. A single, commonly used reference gene, GAPDH, was applied due to its well-documented stability and routine use in qPCR analyses. This method is normalized because it allows the determination of relative changes in gene expression levels [95].

### 4.4. Statistical Analysis

The values of ED_50_ (protecting 50% of mice against tonic convulsions), TD_50_ (50% toxic dose in the chimney test), and the quotient of ED_50_ and TD_50_ (protective index) were assessed for VPA in every protocol of administration.

Both the ED_50_ and TD_50_ values with their respective 95% confidence limits were estimated using computer log-probit analysis according to Litchfield and Wilcoxon (ED_50.EXE) [87]. Subsequently, the standard error (SEM) of the mean values was calculated. The ED_50_ and TD_50_ values were compared using the log-probit method for a single comparison and the unpaired Student’s *t*-test with Welch’s correction [87].

Qualitative variables from the chimney test were compared by Fisher’s exact probability test, whereas the results obtained in the step-through passive avoidance task were statistically evaluated using Kruskal–Wallis nonparametric analysis of variance (ANOVA) followed by the post hoc Dunn’s test.

The plasma and the brain concentrations of VPA were evaluated by the use of one-way analysis of variance (ANOVA) followed by the post hoc Dunnett’s test. The significance level was set at *p* ≤ 0.05 [98].

Statistical analyses of gene expression levels were performed using STATISTICA version 13.3 software (Tibco Corporation, Palo Alto, CA, USA), which enables comprehensive evaluation of quantitative data and their comparison between study groups. The Kruskal–Wallis test, Mann–Whitney U test, and Spearman’s rank correlation coefficient were applied. A *p*-value of < 0.05 was considered the threshold for statistical significance. Cluster analysis was visualized using heat maps generated in the Heatmapper program [99], while correlation analysis was performed using Displayr software (June 2025, Prymont NSW 2009, Australia) [100]. To control the level of statistical significance, Bonferroni correction was applied by appropriately adjusting the calculated significance level. Bonferroni correction was calculated using http://www.policzto.com.pl/index.php/kalkulatory-statystyczne (accessed on 5 May 2026) [101]. In addition, effect sizes were calculated, allowing the strength of observed relationships to be assessed. Effect sizes were determined using https://www.psychometrica.de/effect_size.html (accessed on 5 May 2026) [102]. The functional analysis of the studied genes was performed using the PANTHER v.18.0 classification system software, GO Ontology database (Mus musculus whole genom) [103,104,105].

## 5. Conclusions

In conclusion, the anticonvulsant effects, side effects, and potential drug interactions of VPA may depend on the treatment duration and dosage. Chronic 14-day treatment with VPA administered twice daily enhanced the drug’s antielectroshock effect and increased the risk of motor dysfunction compared with single-dose administration. The observed effects are likely associated primarily with pharmacodynamic mechanisms and accompanied by alterations in the expression of selected genes, including CACNA1G, GAD1, and SCN1A, following chronic administration of the higher VPA dose. In contrast, the lower dose of VPA induced a significant decrease in GABRA1 expression.

These changes in gene expression may reflect adaptive responses of target sites to prolonged drug exposure and could contribute to the observed behavioral effects. However, since neither protein expression nor direct epigenetic parameters were evaluated in the present study, conclusions regarding the precise molecular and epigenetic mechanisms should be interpreted with caution. Further studies are planned to assess the expression of the corresponding protein products and to better characterize the underlying regulatory mechanisms. A strong correlation between gene expression profiles suggests that these genes may be co-regulated and involved in related biological pathways. Additionally, this study highlights the importance of validating findings from acute experiments in long-term protocols and supports the inclusion of chronic drug administration paradigms in preclinical research.

## Figures and Tables

**Table 1:** Effects of acute and chronic treatment with valproate (VPA) on motor performance in the chimney test.

Groups (VPA mg/kg)	Treatment Protocol	Animals Impaired (%)
MW	14 × 2	0
A-1 (VPA 274.8)	1 × 1	0
A-2 (VPA 274.8)	14 × 2	0
B-1 (VPA 231.1)	1 × 1	0
B-2 (VPA 231.1)	14 × 2	0

Data are expressed as the percentage of animals that failed to perform the chimney test. Statistical analysis of data was calculated by using Fisher’s exact probability test. VPA—valproate; treatment protocols: 1 × 1, 1 injection; 14 × 2, 2 injections daily for 14 days.

**Table 2:** Effects of acute and chronic treatment with valproate (VPA) on its protective index in mice.

Treatment Protocol	ED_50_ (mg/kg)	TD_50_ (mg/kg)	PI
VPA 1 × 1	274.8 [253.9–297.4]	465.0 [450.7–480.0]	1.69
VPA 14 × 2	231.1 [205.5–259.8] *	364.1 [333.4–397.7] ***	1.58

Data are expressed as median effective doses (ED_50_s in mg/kg) protecting 50% of the animals against the maximal electroshock-induced seizures and median toxic doses (TD_50_s in mg/kg) inducing motor impairment in 50% of animals tested in the chimney test of VPA with 95% confidence limit in parentheses. The protective index (PI) is a quotient of TD_50_ and ED_50_ values. VPA—valproate; treatment protocols: 1 × 1, 1 injection; 14 × 2, 2 injections daily for 14 days. * *p* < 0.05 vs. VPA 1 × 1; *** *p* < 0.001 vs. VPA 1 × 1.

**Table 3:** Effects of acute and chronic treatment with valproate (VPA) on long-term memory in mice.

Groups (VPA mg/kg)	Treatment Protocol	Retention Time (s)
MW	14 × 2	180 (180; 180)
A-1 (VPA 274.8)	1 × 1	77 (53; 180) *
A-2 (VPA 274.8)	14 × 2	180 (180; 180)
B-1 (VPA 231.1)	1 × 1	180 (180; 180)
B-2 (VPA 231.1)	14 × 2	180 (100; 180)

Data are expressed as median retention time (with 25th and 75th percentiles), during which the animals avoided the dark compartment in the step-through passive avoidance task. Statistical analysis of data was performed using the nonparametric Kruskal–Wallis ANOVA test followed by Dunn’s *post hoc* test. VPA—valproate; treatment protocols: 1 × 1, 1 injection; 14 × 2, 2 injections daily for 14 days; * *p* < 0.05 vs. vehicle.

**Table 4:** Effects of chronic treatment with valproate on its anticonvulsant action in animal seizure models.

Model/Animal	VPA (Dose, Treatment Protocol)	Anticonvulsant Effect	References
MES, PTZ, PCT, BIC	*b.i.d.* for 3 and 5 days	Increased (↓ ED_50_)	[24] *
MEST in mice	500–580 mg/kg/24 h *p. o.* for 12 days	Increased after 4 days	[25]
PTZ *i.v.* in mice	200 mg/kg *b.i.d. i.p.* for 2.5 or 7.5 days	Increased PTZ threshold up to three-fold	[26]
PTZ kindling in mice	300 mg/kg/24 h *i.p.* for 20 days	Reduced seizure severity	[27]
PTZ *i.v.* in rats	200 mg/kg *i.p. q.d.* or *t.i.d.* for 4 days; 200 mg/kg *i.p.* + infusion 400 mg/kg/24 h *i.v.* for 4 days	Increased on the second day of treatment and thereafter (*t.i.d.* or *q.d.* + infusion)	[28]
Amygdala-kindled rats	200 mg/kg *i.p. t.i.d.* (up to 7 doses)	Increased	[29]
	200 mg/kg *i.p. t.i.d.* for 6 weeks	Two-step increase: At day 3 and more permanent after 4 to 6 weeks	[30]
	0.2–0.8 mg/h *i.c.v.* for 7 days	Increased	[31]
	200 mg/kg *i.p. b.i.d.* for 12 days	No tolerance	[32]
	250 mg/kg *p. o.* lub *i.p.* 1 h before stimulation (10 bidaily)	Tolerance to the anticonvulsant effect	[33,34]
	480 µg/day, 720 µg/day, 960 µg/day intrasubthalamic microinfusion for 3 weeks	Long-lasting antiseizure effect	[35]
*n* mutant seizure mice	400 mg/kg/24 h *p. o.* since birth up to 3 months	Reduced seizure frequency and mortality	[36]
Spontaneous recurrent seizures after PILO-induced SE in rats	200 mg/kg *i.p. t.i.d.* for 2 weeks	Indication of tolerance in the second week	[37]
Alumina gel monkey model	Continuous infusion *i.v.* plasma level range 50–150 μg/mL	At the lower plasma levels ↓ seizure frequency for 2 days, permanent effect at the higher plasma levels	[38]
Absence epilepsy in rats	170 mg/kg *i.p. t.i.d.* for 14 days	Signs of tolerance to the drug (confined to the number of spike-wave complexes but not the duration of the discharges) developed from day 5	[39]
Genetically epilepsy-prone gerbils	*s.c.* infusion for 2 weeks, plasma level 40 µg/mL	Moderate anticonvulsant effect (sub-therapeutic drug level)	[40]

BIC—bicuculline, MES—maximal electroshock, MEST—maximal electroshock seizure threshold, PCT—picrotoxin, PILO—pilocarpine, PTZ—pentylenetetrazole, *i.c.v.*—intracerebroventricular, *b.i.d.*—twice a day, *i.p.*—intraperitoneal, *i.v.*—intravenous, *p. o.*—orally, *q.d.*—once a day, *s.c.*—subcutaneous, *t.i.d.*—three times a day, ↓—reduced, ED_50_—median effective dose; * incomplete data.

## Data Availability

The raw data supporting the conclusions of this article will be made available by the authors on request.

## Supplementary Materials

The following supporting information can be downloaded at: https://www.mdpi.com/article/10.3390/ijms27125281/s1.
