# Supplementary material for: The Anticonvulsant Effect of Long-Term Valproate Might Be Attributable to Altered Expression of Selected Genes in Mice
Source: Int J Mol Sci. 2026 Jun 10;27(12):5281. doi: 10.3390/ijms27125281 (PMC13300035; doi:10.3390/ijms27125281)
Supplement: Supplementary file 1 [file ijms-27-05281-s001.zip › ijms-4257759-supplementary.pdf]

Supplementary Materials

The Anticonvulsant Effect of Long-Term Valproate Might Be Attributable to Altered Expression of Selected Genes in Mice

Monika Banach [1,†], Przemysław Kołodziej [2,*,†], Jacek Bogucki [3], Kinga Borowicz [1,*,‡] and Anna Bogucka-Kocka [2,‡]

[1] Independent Experimental Neuropathophysiology Unit, Department of Toxicology, Faculty of Pharmacy, Medical University of Lublin, Jaczewskiego 8b Street, 20-090 Lublin, Poland; monika.banach@umlub.edu.pl

[2] Department of Biology and Genetics, Faculty of Pharmacy, Medical University of Lublin, Chodzki 4A Street, 20-093 Lublin, Poland; anna.bogucka-kocka@umlub.edu.pl

[3] Department of Research Methodology in Medicine, Institute of Medical Biology, Faculty of Medicine, The John Paul II Catholic University of Lublin, Konstantynów 1H St., 20-708 Lublin, Poland; jacek.bogucki@kul.pl

* Correspondence: przemyslaw.kolodziej@umlub.edu.pl (P.K.); kinga.borowicz@umlub.edu.pl (K.B.)

† These authors contributed equally as first authors.

‡ These authors contributed equally as senior authors.

Table S1. List of experimental groups and VPA doses in groups subjected to the MES test. The last dose of VPA was administered 30 min before the test. The percentage of mice protected against maximal electroshock-induced tonic hindlimb extension was assessed in each subgroup consisting of 8 animals. The total number of animals: 310.

Group	Treatment protocol (days x injections)	Doses of VPA in subgroups (mg/kg)	Percentage of mice protected	ED_{50} in mg/kg [95% confidence limits]
1	1 x 1	1A 225<br>1B 250<br>1C 275<br>1D 300	12,5<br>25<br>43,75<br>75	274,8<br>[253,9÷297,4]

	14 x 2	1E 200<br>1F 225<br>1G 250<br>1H 275	25<br>50<br>62,5<br>75	231,1<br>[205,5÷259,8]
2	1 x 1	2A 200<br>2B 225<br>2C 250<br>2D 275	12,5<br>37,5<br>62,5<br>87,5	235,3<br>[217,4÷254,6]
	14 x 1	2E 200<br>2F 225<br>2G 250<br>2H 275<br>2I 300	25<br>37,5<br>37,5<br>62,5<br>75	251,8<br>[220,3÷287,8]
3	1 x 1	3A 225<br>3B 250<br>3C 275	25<br>50<br>87,5	244,6<br>[226,9÷263,7]
	7 x 2	3D 200<br>3E 225<br>3F 250<br>3G 275<br>3H 300	1<br>50<br>50<br>75<br>75	254,6<br>[234,4÷276,6]
4	1 x 1	4A 200<br>4B 225<br>4C 250	6,25<br>50<br>75	230,6<br>[215,4÷247,0]
	7 x 1	4D 200<br>4E 225<br>4F 250	12,5<br>25<br>75	234,7<br>[215,4÷255,8]

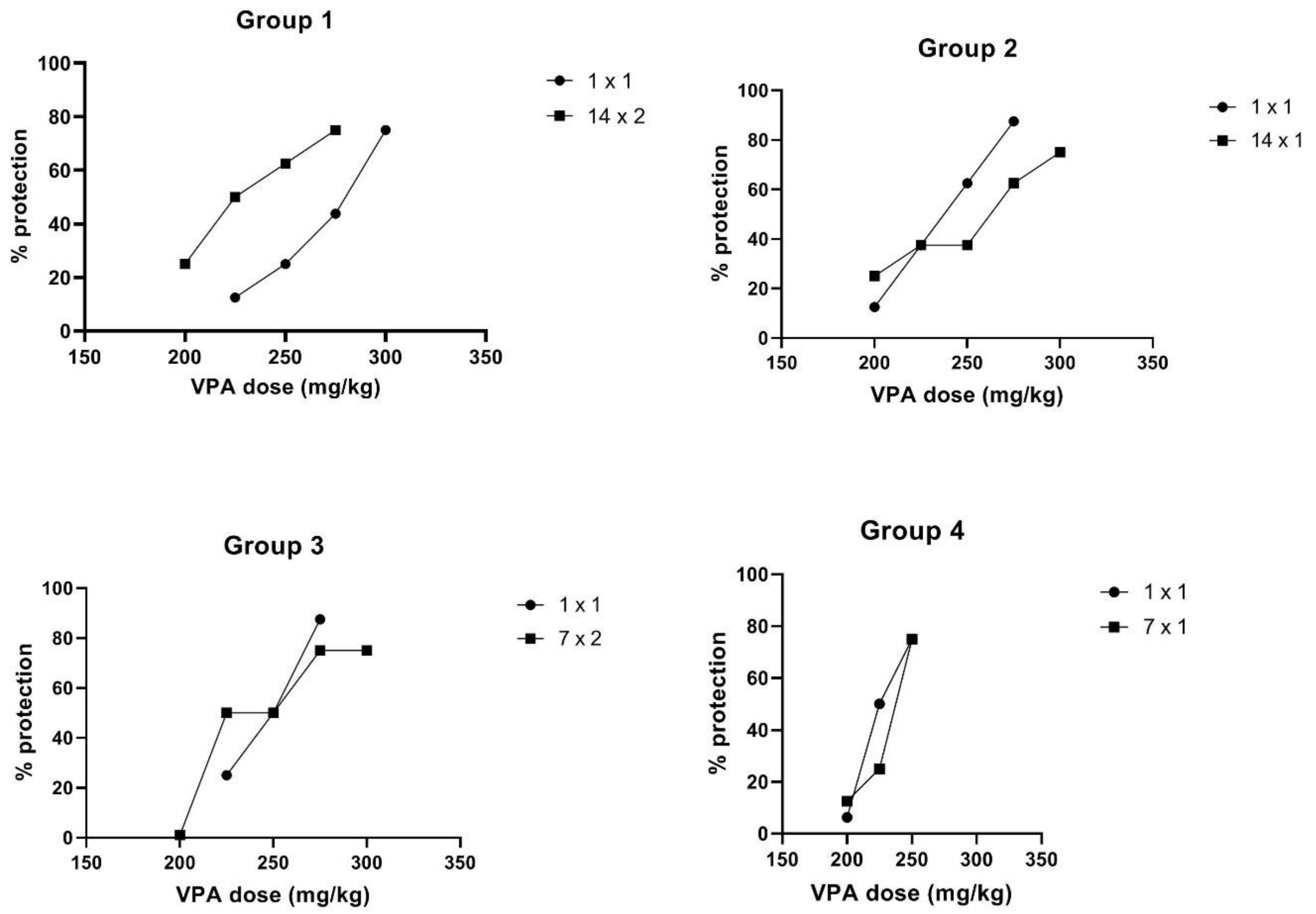

Figure S1. Dose–response relationship curves for the effects of VPA administered intraperitoneally (i.p.) in accordance with four chronic protocols on MES seizures in mice. VPA was administered acutely (1 injection) or chronically 30 min before test; treatment groups: 1 - 2 injections daily for 14 days; 2 - 1 injection daily for 14 days; 3 - 2 injections daily for 7 days; 4 - 1 injection daily for 7 days VPA-valproate, MES-the maximal electroshock.

Table S2. List of experimental groups and VPA doses in groups subjected to the experimental procedures. The last dose of VPA was administered 30 min before the test. The total number of animals: 264.

**Groups and treatment protocol (days x injections)**	**Doses of VPA in groups (mg/kg)**	**Chimney test**	**Step-through passive avoidance task**	**Isolation of the hippocampus**
		**Endpoint assessed**		
A-1 (1 x 1)	274,8	1) % of animals unable to complete the test<br>2) TD_{50}	The time taken to enter the dark box and the median latencies (retention time) with 25th and 75th percentiles	Isolation of mRNA
A-2 (14 x 2)	274,8			
B-1 (1 x 1)	231,1			
B-2 (14 x 2)	231,1			
MW (14 x 2)	Vehicle			
Total number of animals		1)50; 2) 114	50	50

Table S3. List of experimental groups and VPA doses in groups subjected to the pharmacokinetic assessment. The last dose of VPA was administered 30 min before the test. The total number of animals: 50.

**Groups and doses of VPA in groups (mg/kg)**	**Treatment protocol (days x injections)**	**Pharmacokinetics:** concentrations of VPA (µg/ml±SD)	
		plasma	brain
5 (274,8)	(1 x 1)	500,4±88,3	68,68±16,84
6 (274,8)	(7 x 1)	412,4±59,3	66,88±16,47
7 (274,8)	(7 x 2)	392,96±53,48	65,28±11,27
8 (274,8)	(14 x 1)	440,00±76,08	63,28±2,81
9 (274,8)	(14 x 2)	389,12±117,88	51,44±15,7
Total number of animals		50	

Table S4. Descriptive statistics of the expression profile of the studied genes in the hippocampus of mice treated with VPA in both single (A-1, B-1) and chronic (A-2, B-2) treatment protocols. MV – calibrator, control. (Data are presented on a logarithmic scale, LogRQ, log10).

	**Descriptive statistics A1**								
Variable	N	Mean	CI	CI	Median	Min.	Max.	SD	SEM
			-95,00%	95,00%					
LogCACNA1G MOUSE	70	0,491944	0,401158	0,58273	0,53052	-0,20204	1,246991	0,380748	0,045508
LogGABRA1 MOUSE	70	-0,16848	-0,29871	-0,03824	-0,01908	-1,92082	0,783189	0,546183	0,065281
LogGAD1 MOUSE	70	0,242411	0,151246	0,333575	0,345372	-1,06048	0,803047	0,382334	0,045698
LogGRIN2B MOUSE	70	0,646791	0,567557	0,726025	0,639974	-0,09854	1,435382	0,332298	0,039717
LogSCN1A MOUSE	70	0,43523	0,348302	0,522158	0,463442	-0,29243	1,256164	0,364567	0,043574
	**Descriptive statistics A2**								
Variable	N	Mean	CI	CI	Median	Min.	Max.	SD	SEM
			-95,00%	95,00%					
LogRQ CACNA1G MOUSE	70	0,869122	0,817039	0,921205	0,861955	0,38507	1,367039	0,218431	0,026108
LogRQ GABRA1 MOUSE	70	-0,03326	-0,15945	0,092929	0,071375	-1,67778	0,884172	0,529218	0,063254
LogRQ GAD1 MOUSE	80	0,468049	0,3913	0,544798	0,493524	-0,60906	1,065057	0,344878	0,038559
LogRQ GRIN2B MOUSE	70	0,838664	0,777527	0,899801	0,820047	0,35679	1,518856	0,256401	0,030646
LogRQ SCN1A MOUSE	70	0,673844	0,608286	0,739401	0,657662	0,08884	1,381512	0,274943	0,032862

	**Descriptive statistics B1**								
Variable	N	Mean	CI	CI	Median	Min.	Max.	SD	SEM
			-95,00%	95,00%					
LogRQ CACNA1G MOUSE	80	0,058033	-0,08343	0,199494	0,134647	-1,31876	1,480093	0,635669	0,07107
LogRQ GABRA1 MOUSE	80	0,210911	0,016894	0,404928	0,266517	-2,09691	2,310678	0,871833	0,097474
LogRQ GAD1 MOUSE	80	-0,0499	-0,20277	0,102966	0,102031	-2,04576	1,282645	0,686917	0,0768
LogRQ GRIN2B MOUSE	80	0,007807	-0,13794	0,153554	0,100019	-1,42022	1,562804	0,65493	0,073223
LogRQ SCN1A MOUSE	80	-0,01171	-0,1646	0,141173	0,091483	-1,61979	1,483402	0,686999	0,076809
	**Descriptive statistics B2**								
Variable	N	Mean	CI	CI	Median	Min.	Max.	SD	SEM
			-95,00%	95,00%					
LogRQ CACNA1G MOUSE	80	0,213704	0,155224	0,272185	0,210172	-0,2815	0,863501	0,262788	0,029381
LogRQ GABRA1 MOUSE	80	-0,21673	-0,32958	-0,10389	-0,14877	-1,65758	0,729813	0,507071	0,056692
LogRQ GAD1 MOUSE	80	-0,01934	-0,097	0,058319	0,011144	-1,05552	0,633973	0,348971	0,039016
LogRQ GRIN2B MOUSE	80	0,28744	0,208502	0,366378	0,311116	-0,41567	1,146035	0,354715	0,039658
LogRQ SCN1A MOUSE	80	0,200895	0,12827	0,27352	0,200153	-0,54212	0,993392	0,326347	0,036487

Table S5. Comparison of the expression levels of the studied genes in the mouse hippocampus in both single (A-1, B-1) and chronic (A-2, B-2) VPA treatment protocols. A. CACNA1G gene, B. GABRA1 gene, C. GAD1 gene, D. GRIN2B gene, E. SCN1A gene. In this analysis, the multiple comparison method (k=4) was used. The critical significance level for the difference, adjusted using the Bonferroni correction, is p/4, what means 0.05 / 4 = 0.0125. The Bonferroni correction was applied by crossing out the calculated significance level. The Bonferroni correction was calculated using: http://www.policzto.com.pl/index.php/kalkulatory-statystyczne. In addition, the difference effect size was calculated—the results are presented in the tables. The difference effect size was calculated using https://www.psychometrica.de/effect_size.html. Statistically significant differences ($p<0.05$) are highlighted in red.

Dependent variable: LogRQ CACNA1G MOUSE	The 'z' value for multiple comparisons; LogRQ CACNA1G MOUSE<br>Independent (grouping) variable: Dose<br>Kruskal-Wallis Test: H (3, N= 300) =118,0650 p =0,000<br>Eta squared (η^2)= 0.389			
	A1<br>R:164,59	A2<br>R:239,07	B1<br>R:101,51	B2<br>R:109,67
A1		5,079884	4,443070	3,868135
A2	5,079884		9,689551	9,114616
B1	4,443070	9,689551		0,595114
B2	3,868135	9,114616	0,595114	
Dependent variable: LogRQ CACNA1G MOUSE	The p-value for multiple (two-tailed) comparisons; LogRQ CACNA1G MOUSE<br>Independent (grouping) variable: Dose<br>Kruskal-Wallis Test: H (3, N= 300) =118,0650 p =0,000			
	A1<br>R:164,59	A2<br>R:239,07	B1<br>R:101,51	B2<br>R:109,67
A1		0,000002	0,000053	0,000658
A2	0,000002		0,000000	0,000000
B1	0,000053	0,000000		1,000000
B2	0,000658	0,000000	1,000000	

(A)

Dependent variable: LogRQ GABRA1 MOUSE	The 'z' value for multiple comparisons; LogRQ GABRA1 MOUSE<br>Independent (grouping) variable: Dose<br>Kruskal-Wallis Test: H (3, N= 300) =19,10208 p =,0003<br>Eta squared (η^2)= 0.054			
	A1<br>R:136,88	A2<br>R:161,79	B1<br>R:179,09	B2<br>R:123,94
A1		1,699140	2,973471	0,911078
A2	1,699140		1,218607	2,665942
B1	2,973471	1,218607		4,020891
B2	0,911078	2,665942	4,020891	
Dependent variable: LogRQ GABRA1 MOUSE	The p-value for multiple (two-tailed) comparisons; LogRQ GABRA1 MOUSE<br>Independent (grouping) variable: Dose<br>Kruskal-Wallis Test: H (3, N= 300) =19,10208 p =,0003			
	A1<br>R:136,88	A2<br>R:161,79	B1<br>R:179,09	B2<br>R:123,94
A1		0,535757	~~0,017667~~	1,000000
A2	0,535757		1,000000	~~0,046064~~
B1	~~0,017667~~	1,000000		0,000348
B2	1,000000	~~0,046064~~	0,000348	

(B)

Dependent variable: LogRQ GAD1 MOUSE	The 'z' value for multiple comparisons; LogRQ GAD1 MOUSE<br>Independent (grouping) variable: Dose<br>Kruskal-Wallis Test: H (3, N= 310) =73,99543 p =,0000<br>Eta squared (η^2)= 0.0540.24			
	A1<br>R:171,83	A2<br>R:219,31	B1<br>R:122,67	B2<br>R:110,24
A1		3,236439	3,351107	4,198515
A2	3,236439		6,818758	7,695909
B1	3,351107	6,818758		0,877151
B2	4,198515	7,695909	0,877151	
Dependent variable: LogRQ GAD1 MOUSE	The p-value for multiple (two-tailed) comparisons; LogRQ GAD1 MOUSE<br>Independent (grouping) variable: Dose<br>Kruskal-Wallis Test: H (3, N= 310) =73,99543 p =,0000			
	A1<br>R:171,83	A2<br>R:219,31	B1<br>R:122,67	B2<br>R:110,24
A1		0,007262	0,004829	0,000161
A2	0,007262		0,000000	0,000000
B1	0,004829	0,000000		1,000000
B2	0,000161	0,000000	1,000000	

(C)

Dependent variable: LogRQ GRIN2B MOUSE	The ‘z’ value for multiple comparisons; LogRQ GRIN2B MOUSE<br>Independent (grouping) variable: Dose<br>Kruskal-Wallis Test: H (3, N= 300) =115,1427 p =0,000<br>Eta squared (η^2)= 0.379			
	A1<br>R:185,77	A2<br>R:224,65	B1<br>R:89,444	B2<br>R:115,81
A1		2,651497	6,784943	4,927632
A2	2,651497		9,523397	7,666086
B1	6,784943	9,523397		1,922500
B2	4,927632	7,666086	1,922500	
Dependent variable: LogRQ GRIN2B MOUSE	The p-value for multiple (two-tailed) comparisons; LogRQ GRIN2B MOUSE<br>Independent (grouping) variable: Dose<br>Kruskal-Wallis Test: H (3, N= 300) =115,1427 p =0,000			
	A1<br>R:185,77	A2<br>R:224,65	B1<br>R:89,444	B2<br>R:115,81
A1		~~0,048082~~	0,000000	0,000005
A2	~~0,048082~~		0,000000	0,000000
B1	0,000000	0,000000		0,327257
B2	0,000005	0,000000	0,327257	

(D)

Dependent variable: LogRQ SCN1A MOUSE	The 'z' value for multiple comparisons; LogRQ SCN1A MOUSE<br>Independent (grouping) variable: Dose<br>Kruskal-Wallis Test: H (3, N= 300) =80,26092 p =,0000<br>Eta squared (η^2)= 0.261			
	A1<br>R:168,17	A2<br>R:220,19	B1<br>R:105,01	B2<br>R:119,56
A1		3,547345	4,449107	3,424262
A2	3,547345		8,112789	7,087944
B1	4,449107	8,112789		1,060815
B2	3,424262	7,087944	1,060815	

Dependent variable: LogRQ SCN1A MOUSE	The p-value for multiple (two-tailed) comparisons; LogRQ SCN1A MOUSE<br>Independent (grouping) variable: Dose<br>Kruskal-Wallis Test: H (3, N= 300) =80,26092 p =,0000			
	A1<br>R:168,17	A2<br>R:220,19	B1<br>R:105,01	B2<br>R:119,56
A1		0,002335	0,000052	0,003699
A2	0,002335		0,000000	0,000000
B1	0,000052	0,000000		1,000000
B2	0,003699	0,000000	1,000000	

(E)

Table S6. Correlation between the expression level of the studied genes in the mouse hippocampus in both single (A-1, B-1) and chronic (A-2, B-2) treatment protocols with VPA (relative to MW control). A. A-1 vs. MW, B. A-2 vs. MW, C. B-1 vs. MW, D. B-2 vs. MW (p<0.05). Values that are not statistically significant are crossed out. Results after Bonferroni correction. Bonferroni correction was calculated using: http://www.policzto.com.pl/index.php/kalkulatory-statystyczne

Variables	**A1**<br>Spearman's rank correlation<br>BD: pairs removed<br>Correlation coefficients marked with red color are significant at p < 0.05000<br>**Confidence intervals** for the correlation coefficient (only for statistically significant r) are shown below the correlation coefficient (±95%).<br>The **Bonferroni correction** was applied to the correlation coefficient in the calculations. The adjusted level of statistical significance for the correlation coefficient is 0.0125. The adjusted coefficients are indicated in strikethrough of red colour font.				
	LogRQ CACNA1G MOUSE	LogRQ GABRA1 MOUSE	LogRQ GAD1 MOUSE	LogRQ GRIN2B MOUSE	LogRQ SCN1A MOUSE
LogRQ CACNA1G MOUSE					
LogRQ GABRA1 MOUSE	~~0.155~~				
LogRQ GAD1 MOUSE	0.663<br>(0.504-0.778)	0.378<br>(0.153-0.565)			
LogRQ GRIN2B MOUSE	0.761<br>(0.638-0.845)	~~0.090~~	0.753<br>(0.627-0.840)	-	
LogRQ SCN1A MOUSE	0.845<br>(0.759-0.901)	~~0.168~~	0.781<br>(0.666-0.859)	0.762<br>(0.639-0.846)	-

(A)

Variables	**A2**<br>Spearman's rank correlation<br>BD: pairs removed<br>Correlation coefficients marked with red color are significant at p < 0.05000<br>**Confidence intervals** for the correlation coefficient (only for statistically significant r) are shown below the correlation coefficient (±95%).<br>The **Bonferroni correction** was applied to the correlation coefficient in the calculations. The adjusted level of statistical significance for the correlation coefficient is 0.0125. The adjusted coefficients are indicated in strikethrough of red colour font.				
	LogRQ CACNA1G MOUSE	LogRQ GABRA1 MOUSE	LogRQ GAD1 MOUSE	LogRQ GRIN2B MOUSE	LogRQ SCN1A MOUSE
LogRQ CACNA1G MOUSE					
LogRQ GABRA1 MOUSE	~~0.081~~				
LogRQ GAD1 MOUSE	0.536<br>(0.341-0.686)	~~0.203~~			
LogRQ GRIN2B MOUSE	0.604<br>(0.427-0.736)	~~-0.058~~	0.725<br>(0.588-0.821)		
LogRQ SCN1A MOUSE	0.606<br>(0.429-0.737)	~~0.053~~	0.729<br>(0.593-0.824)	0.662<br>(0.503-0.777)	-

(B)

Variables	**B1**<br>Spearman's rank correlation<br>BD: pairs removed<br>Correlation coefficients marked with red color are significant at $p < 0.05000$<br>**Confidence intervals** for the correlation coefficient (only for statistically significant r) are shown below the correlation coefficient (±95%).<br>The **Bonferroni correction** was applied to the correlation coefficient in the calculations. The adjusted level of statistical significance for the correlation coefficient is 0.0125. The adjusted coefficients are indicated in strikethrough of red colour font.				
	LogRQ CACNA1G MOUSE	LogRQ GABRA1 MOUSE	LogRQ GAD1 MOUSE	LogRQ GRIN2B MOUSE	LogRQ SCN1A MOUSE
LogRQ CACNA1G MOUSE					
LogRQ GABRA1 MOUSE	0.702<br>(0.556-0.805)				
LogRQ GAD1 MOUSE	0.835<br>(0.744-0.895)	0.793<br>(0.683-0.867)			
LogRQ GRIN2B MOUSE	0.927<br>(0.883-0.954)	0.728<br>(0.594-0.823)	0.932<br>(0.891-0.957)		
LogRQ SCN1A MOUSE	0.899<br>(0.840-0.936)	0.688<br>(0.537-0.795)	0.896<br>(0.836-0.934)	0.917<br>(0.868-0.948)	

(C)

Variables	**B2**<br>Spearman's rank correlation<br>BD: pairs removed<br>Correlation coefficients marked with red color are significant at p < 0.05000<br>**Confidence intervals** for the correlation coefficient (only for statistically significant r) are shown below the correlation coefficient (±95%).<br>The **Bonferroni correction** was applied to the correlation coefficient in the calculations. The adjusted level of statistical significance for the correlation coefficient is 0.0125. The adjusted coefficients are indicated in strikethrough of red colour font.				
	LogRQ CACNA1G MOUSE	LogRQ GABRA1 MOUSE	LogRQ GAD1 MOUSE	LogRQ GRIN2B MOUSE	LogRQ SCN1A MOUSE
LogRQ CACNA1G MOUSE					
LogRQ GABRA1 MOUSE	~~-0.031~~				
LogRQ GAD1 MOUSE	0.473<br>(0.264-0.639)	~~0.264~~			
LogRQ GRIN2B MOUSE	0.673<br>(0.517-0.785)	~~-0.142~~	0.707<br>(0.563-0.809)		
LogRQ SCN1A MOUSE	0.700<br>(0.554-0.804)	~~0.120~~	0.701<br>(0.555-0.804)	0.675<br>(0.520-0.786)	-

(D)

Table S7. Descriptive statistics of the expression profile of the studied genes in the hippocampus of mice treated with VPA in chronic treatment protocols (A-2, B-2). Data are presented as mean gene expression levels (on a logarithmic scale, LogRQ). SD – standard deviation. A. group A-2 B. group B-2. A-1, B-1 – calibrator, control.

Variable	Descriptive statistics									
	Group	N	Mean	CI -95,00%	CI 95,00%	Median	Min.	Max.	SD	SEM
LogRQ CACNA1G	A2	49	0,381230	0,274369	0,488091	0,407221	-0,282329	0,982181	0,372035	0,053148
LogRQ GABRA1	A2	49	0,138309	0,040906	0,235712	0,099681	-0,586700	0,933740	0,339109	0,048444
LogRQ GAD	A2	56	0,226485	0,141643	0,311327	0,196107	-0,387216	1,039017	0,316810	0,042336
LogRQ GRIN2B	A2	49	0,196972	0,110330	0,283613	0,173478	-0,346787	0,891147	0,301640	0,043091
LogRQ SCN1A	A2	49	0,246531	0,141097	0,351964	0,249687	-0,412289	0,951386	0,367066	0,052438
LogRQ CACNA1G	B2	64	0,157068	-0,004167	0,318303	0,141889	-1,18046	1,614032	0,645477	0,080685
LogRQ GABRA1	B2	64	-0,429420	-0,615506	-0,243334	-0,335902	-2,09691	0,938520	0,744963	0,093120
LogRQ GAD	B2	64	0,027966	-0,135245	0,191177	-0,044733	-1,26761	1,615571	0,653386	0,081673
LogRQ GRIN2B	B2	64	0,275813	0,104987	0,446640	0,270915	-1,24413	1,820615	0,683873	0,085484
LogRQ SCN1A	B2	64	0,208719	0,032377	0,385061	0,179549	-1,25964	1,853510	0,705954	0,088244

Table S8. Comparison of the expression levels of the studied genes in the mouse hippocampus in chronic VPA treatment protocols (A-2, B-2) relative to the corresponding single VPA treatment protocols (A-1 and B-1, respectively). Statistically significant results are highlighted in red. In addition, effect sizes were calculated for statistically significant differences. Coefficient: Cohen's d. https://www.psychometrica.de/effect_size.html

Variable	Mann-Whitney U Test<br>Variable: Group<br>Results marked with an asterisk are statistically significant at p < 0.05000					
	Sum.rang A2	Sum.rang B2	U	Z	p	Z corected
LogRQ CACNA1G	3191,000	3250,000	1170,000	2,305862	0,021119	2,305862
LogRQ GABRA1	3579,500	2861,500	781,500	4,556684	0,000005	4,556712
LogRQ GAD	3873,000	3387,000	1307,000	2,551266	0,010734	2,551274
LogRQ GRIN2B	2677,000	3764,000	1452,000	-0,672060	0,501546	-0,672063
LogRQ SCN1A	2924,500	3516,500	1436,500	0,761861	0,446143	0,761864

Variable	Mann-Whitney U Test<br>Variable: Group<br>Results marked with an asterisk are statistically significant at p < 0.05000				
	p	N A2	N . B2	p	d_{Cohen}
LogRQ CACNA1G	0,021119	49	64	0,020866	0.444
LogRQ GABRA1	0,000005	49	64	0,000003	0.949
LogRQ GAD	0,010733	56	64	0,010460	0.479
LogRQ GRIN2B	0,501544	49	64	0,504883	-
LogRQ SCN1A	0,446141	49	64	0,447651	-

Table S9. Correlation between the expression level of the studied genes in the mouse hippocampus in chronic (A-2, B-2) treatment protocols with VPA (relative to A-1 and B-1control, respectively). A. A-2 vs. A-1, B. B-2 vs. B1 (p<0.05). Values that are not statistically significant are crossed out. Results after Bonferroni correction.

Zmienna	Spearman's rank correlation<br>BD: pairs removed<br>Correlation coefficients marked with red color are significant at p < 0.05000<br>**Confidence intervals** for the correlation coefficient (only for statistically significant r) are shown below the correlation coefficient (±95%).<br>The **Bonferroni correction** was applied to the correlation coefficient in the calculations. The adjusted level of statistical significance for the correlation coefficient is 0.0125. The adjusted coefficients are indicated in strikethrough of red colour font.				
	LogRQ CACNA1G	LogRQ GABRA1	LogRQ GAD	LogRQ GRIN2B	LogRQ SCN1A
LogRQ CACNA1G MOUSE					
LogRQ GABRA1 MOUSE	~~0.283~~				
LogRQ GAD MOUSE	0.871<br>(0.798-0.918)	0.303<br>(0.069-0.504)			
LogRQ GRIN2B MOUSE	0.851<br>(0.768-0.905)	~~0.141~~	0.802<br>(0.696-0.873)		
LogRQ SCN1A MOUSE	0.952<br>(0.923-0.970)	~~0.225~~	0.878<br>(0.808-0.923)	0.914<br>(0.863-0.946)	

(A)

Zmienna	Spearman's rank correlation<br>BD: pairs removed<br>Correlation coefficients marked with red color are significant at p < 0.05000<br>**Confidence intervals** for the correlation coefficient (only for statistically significant r) are shown below the correlation coefficient (±95%).<br>The **Bonferroni correction** was applied to the correlation coefficient in the calculations. The adjusted level of statistical significance for the correlation coefficient is 0.0125. The adjusted coefficients are indicated in strikethrough of red colour font.				
	LogRQ CACNA1G	LogRQ GABRA1	LogRQ GAD1	LogRQ GRIN2B	LogRQ SCN1A
LogRQ CACNA1G MOUSE					
LogRQ GABRA1 MOUSE	0.844<br>(0.758-0.901)				
LogRQ GAD MOUSE	0.936<br>(0.898-0.960)	0.901<br>(0.843-0.937)			
LogRQ GRIN2B MOUSE	0.939<br>(0.902-0.962)	0.791<br>(0.681-0.866)	0.937<br>(0.899-0.960)		
LogRQ SCN1A MOUSE	0.963<br>(0.940-0.977)	0.837<br>(0.747-0.896)	0.940<br>(0.904-0.962)	0.951<br>(0.921-0.969)	

(B)
